# Genome-wide expression analysis of soybean NF-Y genes reveals potential function in development and drought response

**DOI:** 10.1007/s00438-014-0978-2

**Published:** 2014-12-27

**Authors:** Truyen N. Quach, Hanh T. M. Nguyen, Babu Valliyodan, Trupti Joshi, Dong Xu, Henry T. Nguyen

**Affiliations:** 1 Division of Plant Sciences and National Center for Soybean Biotechnology, University of Missouri, Columbia, MO 65211 USA; 2 Present Address: Field Crop Research Institute, Vietnam Academy of Agricultural Sciences, Hanoi, Vietnam; 3 Present Address: The Center for Plant Science Innovation, University of Nebraska, Lincoln, NE USA; 4 Department of Computer Science, Christopher S. Bond Life Sciences Center, National Center for Soybean Biotechnology and Informatics Institute, University of Missouri, Columbia, MO USA

**Keywords:** NF-Y, Nodulation, Transcription factor, Soybean, Water stress

## Abstract

Nuclear factor-Y (NF-Y), a heterotrimeric transcription factor, is composed of NF-YA, NF-YB and NF-YC proteins. In plants, there are usually more than 10 genes for each family and their members have been identified to be key regulators in many developmental and physiological processes controlling gametogenesis, embryogenesis, nodule development, seed development, abscisic acid (ABA) signaling, flowering time, primary root elongation, blue light responses, endoplasmic reticulum (ER) stress response and drought tolerance. Taking the advantages of the recent soybean genome draft and information on functional characterizations of nuclear factor Y (NF-Y) transcription factor family in plants, we identified 21 *GmNF*-*YA*, 32 *GmNF*-*YB*, and 15 *GmNF*-*YC* genes in the soybean (*Glycine max*) genome. Phylogenetic analyses show that soybean’s proteins share strong homology to *Arabidopsis* and many of them are closely related to functionally characterized NF-Y in plants. Expression analysis in various tissues of flower, leaf, root, seeds of different developmental stages, root hairs under rhizobium inoculation, and drought-treated roots and leaves revealed that certain groups of soybean NF-Y are likely involved in specific developmental and stress responses. This study provides extensive evaluation of the soybean NF-Y family and is particularly useful for further functional characterization of GmNF-Y proteins in seed development, nodulation and drought adaptation of soybean.

**Electronic supplementary material:**

The online version of this article (doi:10.1007/s00438-014-0978-2) contains supplementary material, which is available to authorized users.

## Introduction

Nuclear factor Y (NF-Y) is a transcription factor (TF) complex that binds the CCAAT element to regulate gene expression. There are three distinct subunits: NF-YA, NF-YB and NF-YC, which contains evolutionary conserved domains for DNA binding and subunit interaction to form the heterotrimeric NF-Y complex. Subunit assembly is well studied in animals and is conserved in plants (Romier et al. 2003; Calvenzani et al. 2012; Hackenberg et al. 2012). Initially, NF-YB and NF-YC form a dimer complex in cytoplasm and then recruit the third subunit, NF-YA, to form a mature heterotrimemeric NF-Y complex TF (Frontini et al. 2004; Kahle et al. 2005). The binding of NF-Y can either activate or repress transcription of genes (Ceribelli et al. 2008). Sequence analysis of mammalian genome showed 25–30 % of the genes having NF-Y binding sites in their promoters (Mantovani 1998). The CCAAT box is typically found in both forward and reverse orientations between 60 and 100 bp upstream of transcription start site in mammalian genes and may be present in multiple copies with variable distances (Mantovani 1998). In a survey of a promoter sequence population, the CCAAT box element appeared highly associated with TATA-less promoters (Mantovani 1998).

In contrast to animals and yeast where there is a single gene encoding each subunit (Mantovani 1999), there are about 10 genes encoding each subunit in plants (Stephenson et al. 2007; Thirumurugan et al. 2008; Siefers et al. 2009; Liang et al. 2013) with temporal, spatial, universal and organ-specific expression patterns associating with various developmental and environmental response regulations. The roles of certain individuals and NF-Y complexes have been reported in regulation of embryogenesis, cellular signaling, flowering time control, nodulation and nitrogen nutrition and stress tolerance. *Arabidopsis* NF-YA genes NF-YA1, 3, 5, 6, 8, and 9 have been known to regulate gametogenesis, embryogenesis, seed morphology, and seed germination (Fornari et al. 2013; Mu et al. 2013). Specific members of NF-YB, particularly the LEC 1 group, have been reported to regulate embryogenesis and seed development (West et al. 1994; Parcy et al. 1997; Hawkins and Nakamura 1999; Kwong et al. 2003). LEC1 functions as an integrator of various regulatory events, involving light and hormone signaling, specifically during somatic and early zygotic embryogenesis (Petroni et al. 2012; Laloum et al. 2013) and fatty acid biosynthesis (Mu et al. 2008; Shen et al. 2010; Tan et al. 2011; Mendes et al. 2013). The third subunit, NF-YC, is also important in many developmental regulations. *Arabidopsis* NF-YC3, NF-YC4 and NF-YC9 physically interact in vivo with both NF-YB2 and NF-YB3 and are required to regulate flowering time.

NF-Y proteins have been reported as a key factor regulating nodulation in the nitrogen-fixing plants and nitrogen assimilation in various plant species. The involvement in nodulation and nitrogen assimilation was identified by the early works on model legume *Medicago truncatula* for MtNF-YA1 (MtHAP2-1) which controls nodule meristem function (Combier et al. 2006). MtNF-YA1 controls infection thread progression from initial root infection through colonization of nodule tissues (Laporte et al. 2013). Later works on *Lotus japonicus* identified two NF-Y subunit genes, LjNF-YA1 and LjNF-YB1, which are targeted by nodule inception (NIN) and acted downstream of NIN in the nodule development regulation (Soyano et al. 2013). These two genes are expressed in the root nodule primordia and their protein product can form a NF-Y complex in plant cells. Knockdown of *LjNF*-*YA1* inhibited root nodule organogenesis and ectopic expression of the *LjNF*-*YA1* and *LjNF*-*YB1* genes caused abnormal cell division during lateral root development, indicating that the Lotus NF-Y subunits can function to induce cortical cell division to initiate root nodule organogenesis. Recently, the C subunit from common bean (*Phaseolus vulgaris*) NF-YC1 was reported to play a key role in the improved nodulation seen by more efficient strains of rhizobia. RNAi-induced reduction of NF-YC1 in hairy roots resulted in the arrest of nodule development and defects in the infection process and the mechanisms appears to involve suppression of cortical cell divisions (Zanetti et al. 2010). Whether PvNF-YC1 functions independently or in combination with other subunits of NF-Y complex, however, has not been known.

In addition to the roles in plant growth and development, NF-Y proteins are important regulators of stress tolerance, especially in response to drought stress (Petroni et al. 2012; Laloum et al. 2013). Overexpression of AtNF-YA5, a strongly drought-induced gene with strong expression in the guard cells, improves drought tolerance and reduces water loss in the leaves (Li et al. 2008). In contrast to the overexpression of AtNF-YA5, the nfya5 knockout plants and plants overexpressing miR169a showed enhanced leaf water loss and were more sensitive to drought stress than wild-type plants. In soybean, GmNF-YA3 is induced by various stress treatments. The *Arabidopsis* transgenic plants overexpressing GmNF-YA3 show reduced leaf water loss and enhanced drought tolerance through regulating the common drought-responsive genes (Ni et al. 2013). In rice, the transgenic plants overexpressing HAP2E (NF-YA), a gene responsive to wounding, are tolerant to the pathogen *Xanthomonas oryzae* pv. *oryzae*, drought and salinity stresses, and improve photosynthesis and tiller numbers (Alam et al. 2014). Drought tolerance regulation is also reported in NF-YB subgroup. Overexpression of the drought-inducible NF-YB results in improved performance under drought conditions in *Arabidopsis*, maize and poplar (Nelson et al. 2007; Han et al. 2013). The transgenic plants have significant improvement in number of stress-related parameters, including chlorophyll content, stomatal conductance, leaf temperature, reduced wilting, maintenance of photosynthesis and the seed yield under water-limited conditions (Nelson et al. 2007). Further characterizations of drought-inducible NF-Y genes will facilitate genetic engineering for improvement of drought tolerance in crop plants.

Soybean is one of the most important crops for its nutrient sources for human and animal consumption, and potential biofuel and economic values. Extensive research has focused on oil seed development (Severin et al. 2010) and plant and symbiotic microbe interaction (Libault et al. 2010), making soybean an ideal plant species for both basic and applied research. Taking the advantage of broad understanding of the recent functional genomics of plant NF-Y genes and the current draft of soybean genome (Schmutz et al. 2010), we attempted to characterize the soybean NF-Y gene family. Together with analysis of NF-Y family in growth and development, we included an analysis of the family members in response to drought and *Bradyrhizobium japonicum* treatments which are particularly important to identify candidate genes involved in drought tolerance and nodulation and nitrogen fixation in soybean. Our study of transcript expression of soybean NF-Y gene families, therefore, would provide an initial step in understanding gene function that may facilitate functional characterization and genetic engineering to increase the efficiency of soybean in response to drought and *B. japonicum*. In this study, we identified and investigated 68 NF-Y (21 GmNF-YA, 32 GmNF-YB, 15 GmNF-YC) and 13 NC2 genes for their expression patterns in soybean tissues and in response to drought stress. Expression analysis showed that there are strong associations between gene expression patterns and homology-based predicted functions, allowing us to assign putative functions to the individual gene groups in regulation of plant growth, development and adaptation to drought stress.

## Materials and methods

### In silico identification of soybean NF-Y family members

All soybean protein sequences were downloaded from the Phytozome biomart, gene model V9.1 (http://www.phytozome.net/search.php) using PFAM ID PF02045 (CBFB/NFYA) and PF00808 (CBFD_NFYB_HMF) and KOG ID KOG0869, KOG0871, KOG1561, KOG1657 and KOG1659, for CCAAT-binding factor subunit A (HAP2), CCAAT-binding factor subunit B (HAP3), CCAAT-binding factor subunit C (HAP5), Class 2 transcription repressor NC2β subunit (Dr1) and Class 2 transcription repressor NC2α subunit (DRAP1), as queries. We used the updated classification (Petroni et al. 2012) of plant NF-Y, NC2 (DR1 and DRAP1) and Dpb3/Dpb4 from *Arabidopsis*, rice and wheat (both full-length and domain sequences) to classify the retrieved soybean proteins. This updated functional classification has corrected some of the previously identified genes in *Arabidopsis*, wheat and rice (Stephenson et al. 2007; Thirumurugan et al. 2008; Siefers et al. 2009) by re-grouping some of the previously annotated NF-YB and NF-YC genes to NC2β, NC2α and Dpb3/Dpb4 (Petroni et al. 2012). Local BLAST was performed with a threshold of E-10 to classify individuals into functional groups.

Retrieved sequences were aligned using CrustalW with a gap open cost of 10.0 and gap extension cost of 1.0. Phylogenetic tree was constructed by MEGA5 software (Tamura et al. 2011) using neighbor-joining method (Poisson model with uniform rates among sites) and the relationship was estimated using a bootstrap analysis with 1,000 replicates. MultiExperiment Viewer TM4 software (Aryee et al. 2009) was used to generate heat map and hierarchical clustering analysis results.

### Plant materials and drought treatment

Soybean cultivar Williams 82 was grown in greenhouse (28/20 °C day/night temperature, 14-h day-length photoperiod, 800 µmol m^−2^ s^−1^ light intensity and 60 % humidity) with a density of four plants per 1-gallon pot, containing a mixture of 1 sand:1 turface. The plants were watered every 2 days. Drought treatments started when the plants reached the first leaf stage (V1—first fully open trifoliolate leaf stage, day 14th) by withholding watering and the plants were harvested when the desired stem water potential was reached. There were three drought treatments with stem water potentials of −0.5 (day 18th), −1.0 (day 22th) and −1.5 MPa (day 34th) for drought and the corresponding control treatments had a stem water potential of ~−0. 2 MPa. To measure stem water potential, the stems were cut at the hypocotyl segment and immediately measured for water potential using a pressure chamber (PMS Instrument Co. Albany, CA, USA) at around 4am. Predawn tissue collection was selected because it was considered equivalent to whole soil water potentials. Three biological replicate experiments were conducted following a complete randomized design. Each replicate consisted of samples pooled from three plants.

### RNA isolation, cDNA synthesis and mRNA expression analyses for drought responses

Each frozen tissue collected from the three-biological repeat drought experiment was separated into two technical repeats and grinded in liquid nitrogen. RNA was extracted using Trizol (Invitrogen Inc.). RNA integrity was verified by electrophoresis using 1 % agarose in buffer TAE. The RNA was purified from DNA contamination using the Turbo DNA-free DNaseI kit (Ambion, Austin, TX, USA). One microgram of total RNA and the primer mix of oligodT and random hexamer were used to synthesize cDNA using iScript cDNA Synthesis Kit (Bio-Rad, Hercules, CA, USA) in a reaction volume of 20 µL. Quantitative real-time PCR (qRT-PCR) was performed to quantify mRNA expression under drought conditions. Initially, PRIMEGENS (Srivastava and Xu 2007) was used to design primers for soybean NF-Y gene family and all the obtained sequences were manually verified for specific alignment by Blast against the soybean genome database housed in Phytozome to have the final primer list (Supplementary Table S2). Primer pairs with acceptable PCR efficiency and the unique melting disassociation curve were used for qRT-PCR. All reference genes (Supplementary Table S3) were selected based on recommendations from current literatures (Jian et al. 2008; Libault et al. 2008; Hu et al. 2009; Le et al. 2012). qRT-PCR results were analyzed using a common PCR efficiency for each primer obtained from linregPCR analysis software (Ramakers et al. 2003). The obtained efficiencies were consistently higher than 1.8 with average of about 1.95 for whole primer sets. GeNorm (Vandesompele et al. 2002) was used to test the stability (M index) to determine a set of reference genes to be used to normalize individual expression data. In our experiment, we calculated relative expression by normalizing data to the four best reference genes: IDE, UNK1, UNK2 and ACT (Supplementary Figure S1). Fold change of gene expression was calculated only when there was a significant difference in means of relative gene expression between the drought and control samples from a Student *T* test.

### Tissue-specific expression and responses to *B. japonicum* treatments

Data were retrieved from previously reported datasets (Libault et al. 2010; Severin et al. 2010) and detailed experimental procedures were described therein and briefly summarized below. Tissue-specific expression in 14 tissues was performed on soybean genotype P-C609-45-2-2 (Severin et al. 2010). The soybean plants were growing in pots containing *B. japonicum*-inoculated soil and full-nutrient fertilizer (Osmocote 14-14-14) in growth chambers under photoperiod of 14/10 h, thermocycle of 22/10 °C, relative humidity of 50–60 %, and light intensity of 550–740 μE m^−2^ s^−1^. Samples were pooled from a minimum of three plants. Young leaf tissue samples were collected at flowering stage and pods and seeds were harvested by seed weight and pod lengths. The 1-cm pod was approximately harvested at 7 days after flowering (DAF), and the 4- and 5-cm pods were harvested at 10–13 DAF and 14–17 DAF, respectively, and were divided into seed and pod-shell components. Seed 21-, 25-, 28- and 35-DAF had seed weights between 10 and 25 mg, 25 and 50 mg, 50 and 100 mg, 100 and 200 mg, and greater than 200 mg, respectively. Root and nodule tissues were from plants grown in growth chambers under 16-h photoperiods and light intensities between 310 and 380 μE m^−2^ s^−1^. Root tissue was harvested after 12 days and nodules were harvested at 20–25 days after bacterial inoculation. For analysis of gene expression in root hairs in response to *B. japonicum* treatments, normalized data were retrieved from a previous report (Libault et al. 2010) and the experimental procedures are described briefly below. At least three independent biological replicates were produced to ensure the reproducibility of the plant tissues analyzed. Soybean (cv. Williams 82) seeds were germinated on nitrogen-free B&D agar medium in growth chambers (dark conditions, 80 % humidity, 27 °C). Three days after sowing, a *B. japonicum* strain USDA 110 cell suspension or water (mock inoculation) was sprayed on the seedlings and the treated seedlings were returned to the original growth chamber for incubation. Root hair cells were isolated after 12, 24 and 48 h of bacterial inoculation. For each condition, similar quantities of total RNA isolated from three independent biological replicates were pooled together to synthesize cDNA which were then ligated to Solexa adaptors. Complementary DNAs were sequenced using Solexa platform and data were analyzed using Illumina Genome Analyzer II, and the data were analyzed and normalized to reads per kilobase per million (RPKM).

## Results

### Identification of NF-Y genes from soybean

BLAST searches using functionally characterized NF-Y domains as queries resulted in 134 sequences belonging to 81 gene loci. We used the primary transcript as a representative from each gene for further analysis of transcriptome. The final gene list is divided into four groups: 21 GmNF-YA, 32GmNF-YB, 15GmNF-YC, 11 NC2 and 2 Dpb3 proteins (Table 1). BLAST searches using *Arabidopsis* protein sequences as queries resulted in similar classified groups (Supplementary Table S1). NC2 and Dpb3 proteins share the histone fold motif (HFM), but lack of essential amino acids for subunit interactions (as discussed later); therefore, were excluded from further functional analysis relating to NF-Y gene families.

**Table 1:** NF-Y and NC2/Dpb genes in soybean

GmNC2/GmDpb			GmNF-YA			GmNF-YB			GmNF-YC		
Name	Primary ID	Characterized proteins	Name	Primary ID	Characterized proteins	Name	Primary ID	Characterized proteins	Name	Primary ID	Characterized proteins
GmNC2β1	Glyma05g07750.1	OsDR1	GmNF-YA01	Glyma02g35190.4	AtNF-YA2	GmNF-YB01	Glyma02g17310.1	TaNF-YB3	GmNF-YC01	Glyma02g09867.1	AtNF-YC1
GmNC2β2	Glyma06g23234.1	OsDR1	GmNF-YA02	Glyma02g47380.5	AtNF-YA9	GmNF-YB02	Glyma02g46970.1	AtNF-YB2	GmNF-YC02	Glyma03g39911.1	AtNF-YC9
GmNC2β3	Glyma17g13260.1	OsDR1	GmNF-YA03	Glyma03g36140.5	AtNF-YA2	GmNF-YB03	Glyma03g18670.2	AtNF-YB6	GmNF-YC03	Glyma04g37291.1	AtNF-YC1
GmNC2β4*	Glyma18g22896.1	OsDR1	GmNF-YA04	Glyma05g29970.1	AtNF-YA4	GmNF-YB04	Glyma03g22721.1	TaNF-YB3	GmNF-YC04	Glyma06g17780.1	AtNF-YC1
GmNC2α1	Glyma02g44500.2	OsDRAP1	GmNF-YA05	Glyma07g04050.9	AtNF-YA3	GmNF-YB05	Glyma03g33490.1	ZmNF-YB2	GmNF-YC05	Glyma08g15700.1	AtNF-YC9
GmNC2α2	Glyma06g04161.1	OsDRAP1	GmNF-YA06	Glyma08g13090.1	AtNF-YA4	GmNF-YB06	Glyma05g31681.1	OsHAP3H	GmNF-YC06	Glyma08g17630.1	MtNF-YC2
GmNC2α3	Glyma06g46850.1	OsDRAP1	GmNF-YA07	Glyma08g45030.1	AtNF-YA1	GmNF-YB07	Glyma05g32680.1	AtNF-YB3	GmNF-YC07	Glyma10g29691.1	AtNF-YC9
GmNC2α4	Glyma13g25860.1	OsDRAP1	GmNF-YA08	Glyma09g02770.2	AtNF-YA4	GmNF-YB08	Glyma07g29695.1	AtNF-YB6	GmNF-YC08	Glyma12g34510.1	AtNF-YC1
GmNC2α5	Glyma14g04321.1	OsDRAP1	GmNF-YA09	Glyma09g07960.4	AtNF-YA3	GmNF-YB09	Glyma07g37840.2	AtNF-YB2	GmNF-YC09*	Glyma13g27770.2	MtNF-YC2
GmNC2α6	Glyma15g36170.1	OsDRAP1	GmNF-YA10	Glyma10g10240.2	AtNF-YA2	GmNF-YB10	Glyma07g39820.1	AtNF-YB6	GmNF-YC10	Glyma13g27780.2	MtNF-YC2
GmNC2α7*	Glyma19g41280.1	OsDRAP1	GmNF-YA11	Glyma12g36540.6	AtNF-YA9	GmNF-YB11	Glyma08g00330.2	AtNF-YB3	GmNF-YC11	Glyma13g27790.1	MtNF-YC2
GmDpb3-1	Glyma11g37130.1	OsDRAP1	GmNF-YA12	Glyma13g16770.1	AtNF-YA3	GmNF-YB12	Glyma08g14931.1	AtNF-YB3	GmNF-YC12	Glyma13g35980.1	AtNF-YC1
GmDpb3-2	Glyma18g01040.1	OsDRAP1	GmNF-YA13	Glyma13g27230.4	AtNF-YA9	GmNF-YB13	Glyma08g44140.2	TaNF-YB3	GmNF-YC13	Glyma15g41486.1	MtNF-YC2
			GmNF-YA14	Glyma14g01360.1	AtNF-YA9	GmNF-YB14	Glyma09g01650.1	TaNF-YB3	GmNF-YC14*	Glyma19g42460.1	AtNF-YC9
			GmNF-YA15	Glyma15g03175.1	AtNF-YA3	GmNF-YB15	Glyma09g05150.2	AtNF-YB2	GmNF-YC15	Glyma20g37620.1	AtNF-YC9
			GmNF-YA16	Glyma15g13660.2	AtNF-YA4	GmNF-YB16	Glyma09g28671.1	AtNF-YB1			
			GmNF-YA17	Glyma15g18970.1	AtNF-YA3	GmNF-YB17	Glyma10g02480.1	AtNF-YB2			
			GmNF-YA18	Glyma16g00711.1	AtNF-YA3	GmNF-YB18	Glyma10g05606.1	ZmNF-YB2			
			GmNF-YA19	Glyma17g05920.1	AtNF-YA3	GmNF-YB19	Glyma10g29440.2	AtNF-YB3			
			GmNF-YA20	Glyma18g07890.2	AtNF-YA1	GmNF-YB20	Glyma10g33550.2	AtNF-YB1			
			GmNF-YA21	Glyma19g38800.2	AtNF-YA2	GmNF-YB21	Glyma11g18190.1	AtNF-YB3			
						GmNF-YB22*	Glyma11g29866.1	ZmNF-YB2			
						GmNF-YB23	Glyma13g10690.2	ZmNF-YB2			
						GmNF-YB24	Glyma15g12570.1	AtNF-YB3			
						GmNF-YB25	Glyma15g16460.2	AtNF-YB1			
						GmNF-YB26	Glyma17g00950.1	AtNF-YB6			
						GmNF-YB27	Glyma17g02810.1	AtNF-YB1			
						GmNF-YB28	Glyma18g08620.1	TaNF-YB3			
						GmNF-YB29*	Glyma19g36220.2	ZmNF-YB2			
						GmNF-YB30	Glyma20g00240.1	AtNF-YB6			
						GmNF-YB31*	Glyma20g34050.2	AtNF-YB1			
						GmNF-YB32	Glyma20g37870.2	TaNF-YB3			

The primary ID was retrieved from Phytozome V9.1 (http://www.phytozome.net/search.php)

The sequences marked with an asterisk (*) were missing the 5′end. The characterized proteins having highest homology to the soybean proteins in alignment analyses are included for reference

For verification purposes, soybean NF-Y protein sequences were downloaded from three current databases of soybean transcription factors. The first database, SoyDB (http://soykb.org/), provides the predicted transcription factors from InterProScan searches against 11 databases integrated in InterPro to find transcription factor domains (Wang et al. 2010). The second soybean transcription database, SoybeanTFDB (http://soybeantfdb.psc.riken.jp/), used Hidden Markov Model (HMM) of PFAM to search against the modeled proteome data of annotated genes in Glyma1 from Phytozome database with a threshold of *E* < 1e-5 and a verification from domain searches using InterProScan and Blastp for putative homologous genes (Mochida et al. 2010). The third transcription factor database, PlantTFDB (http://planttfdb.cbi.pku.edu.cn/), used 48 HMM-Pfam profiles and 16 additional HMM constructed from GenPept and Swiss-Prot protein databases to construct 64 TF families in *Arabidopsis* and other plant species (Guo et al. 2008). Alignment analysis of these obtained sequences resulted in a number of genes incorrectly classified to NF-YB and NF-YC subfamilies (data not shown) mainly due to the fact that the classification did not have a separate description and classification for NF-YB, NF-YC, NC2 or Dpb group, but instead, have a common description for these two groups with a name NF-YB/HMF domain. Given the critical differences among NC2, Dpb and NF-Y groups, it is necessary to annotate them accurately for the updated research.

### Tissue differential expression and drought and *B. japonicum* inducibility of NF-Y genes

**Fig. 1:**
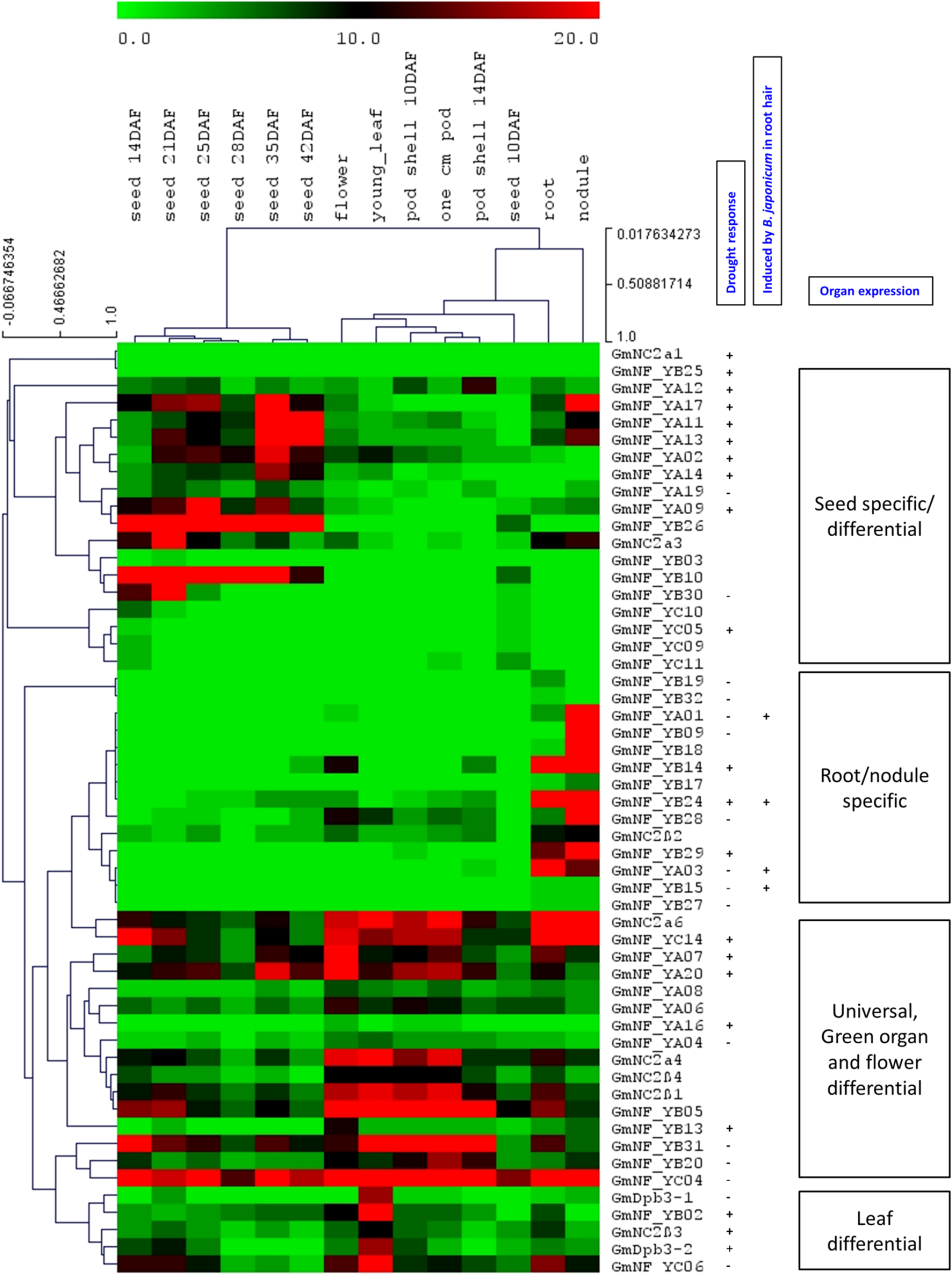
Expression of soybean NF-Y in various tissues, drought stress and rhizobium inoculation. Data of 54 GmNF-Y genes were retrieved from the normalized RNA sequencing databases (Libault et al. 2010; Severin et al. 2010) for clustering analysis of tissue-specific and stress-response expression. For drought and rhizobium treatments (+) and (–) indicate up and down gene expression in both root and leaf tissues tested, compared to non-stress condition. The *color bar* represents number of RPKM (color figure online)

Publicly available gene expression in 14 soybean tissue types including leaf, flower, pod, root, nodules and various stages of seed development (Severin et al. 2010) for 54 soybean NF-Y genes was retrieved and used to construct a hierarchical clustering heat map (Fig. 1). The data show that there are some preferences in expression of each gene subfamily in certain plant tissues. A small group of genes highly specific to 1- to 3-week-old seeds (R3 growth-staged seeds) belong strictly to NF-YC (GmNF-YC05, GmNF-YC09, GmNF-YC10 and GmNF-YC11) while majority of seed deferential genes with stronger expression belong to NF-YA and NF-YB genes. A large group of 14 genes strictly to NF-YA and NF-YB are highly specific to root and/or nodules. We also included the data retrieved from a mRNA expression analysis of root hairs in response to treatments of *B. japonicum* (Libault et al. 2010) which clearly show that all the genes that are strongly induced (10- to 63-fold change) by *B. japonicum* in root hair are nodule-specific GmNF-YA members: NF-YA01, NF-YA03, NF-YA10 and NF-YA21 (Fig. 1; Supplementary Figure S2). There are several smaller gene groups which are more universal in expression and can be sub-grouped due to slight preference in green organs, flowers and leaves. Members of this group belonging to all families of NF-Y and NC2 including NF-YC14, NF-YB13, NF-YA07 and NF-YA20 are differentially expressed in green organs (leaves and pods) while NF-YB02, NF-Y06, Dpb3-1, Dpb3-2 and NC2β3 differentially expressed in leaves.

**Fig. 2:**
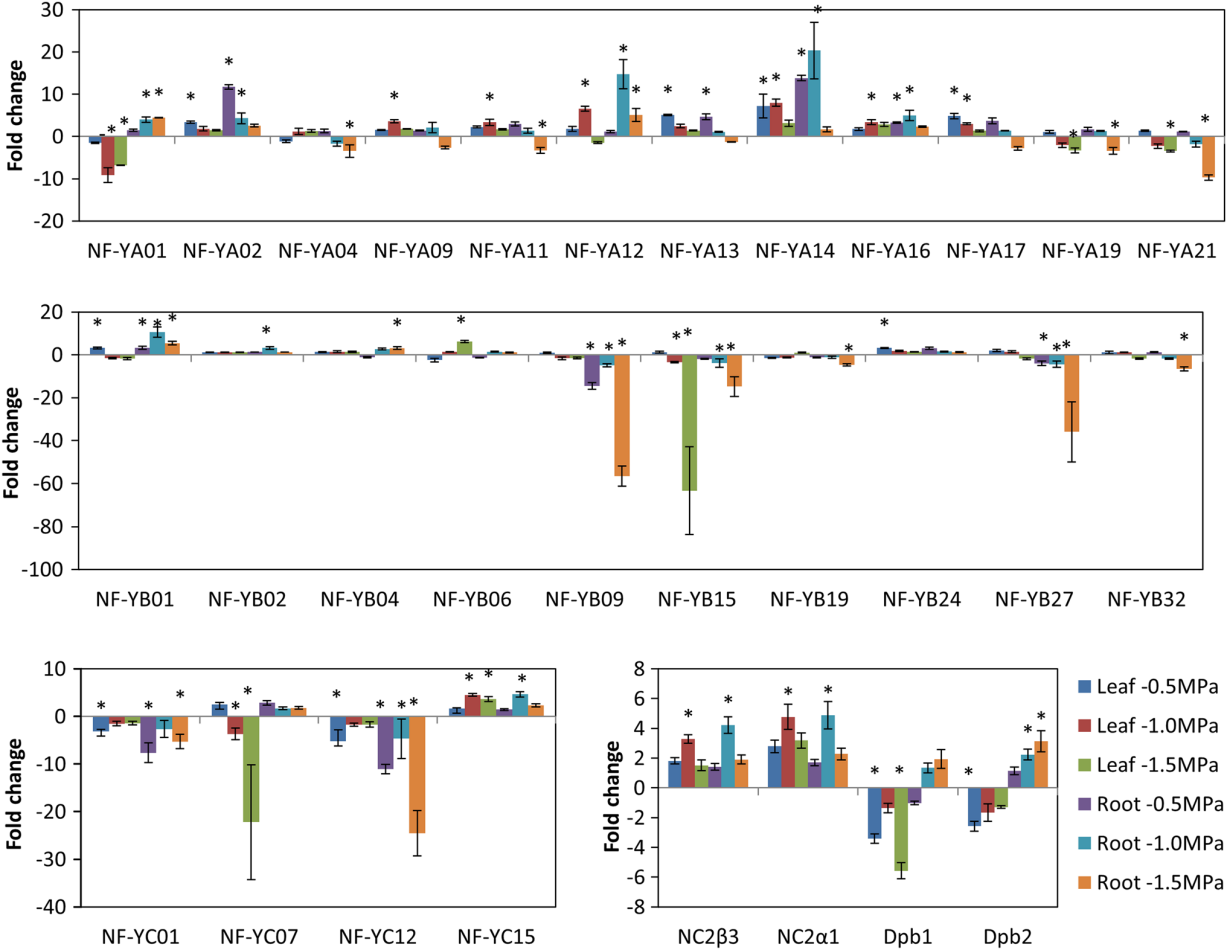
Expression of highly drought-responsive soybean NF-Y genes. These genes have at least threefold-changed (FC) expression compared to the non-stress controls. Significant FCs were marked with an *asterisk* from three biological repeats

To identify candidate genes for drought tolerance in soybean, we performed transcript profiling for the soybean CCAAT box gene family using qRT-PCR for root and leaf samples of three drought treatments of −0.5, −1.0 and −1.5 MPa predawn stem water potentials. We were able to amplify and quantify expression of total 75 genes (Supplementary Table S2). Using a fold-change cut-off of three, 30 genes were found to respond to drought treatment in soybean leaf and root samples (Fig. 2) and NF-YA members appear to be more drought responsive. Most seed differentially expressing genes are drought induced while the root/nodule-specific genes have tendency to be drought repressed (Fig. 1).

### Domain structure and putative function of soybean NF-Y subunits

#### GmNF-YA

**Fig. 3:**
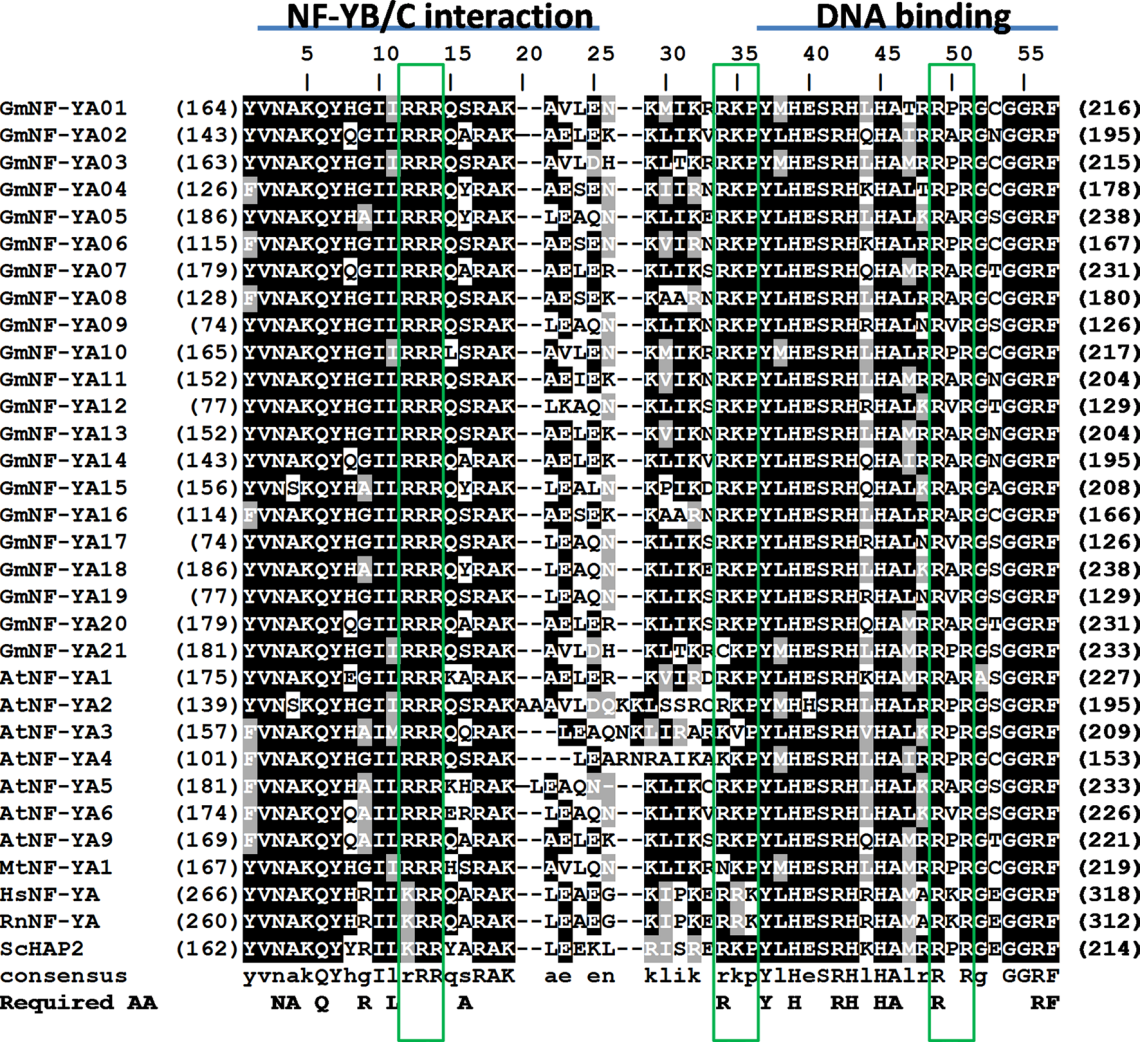
Alignment of soybean NF-YA domains. The referred sequences are from *Arabidopsis* (At), *Medicago* (Mt), human (Hs), mouse (Rn) and yeast (Sc). *Numbers in parentheses* are the actual amino acid numbers of the start and end of NF-YA domain. Required amino acids (Required AA) are important for DNA binding and subunit interaction from yeast (Xing et al. 1993) and rat (Maity and de Crombrugghe 1992). The *three boxes in green* are the basic residual clusters required for nuclear targeting (Peng et al. 1998) (color figure online)

Using the sequences of known NF-YA/HAP2 proteins, we could identify 21 GmNF-YA genes from the soybean genome. Soybean NF-YA proteins have variable length from 206 to 347 amino acids, a comparable range with *M. truncatula* (Laloum et al. 2013). There is no similarity between NF-YA and other transcription families except some homology to the CCT domain of the flowering time regulator CONSTANS (Wenkel et al. 2006). NF-YA proteins are characterized by a conserved sequence that includes a protein interaction domain which can bind to the combined surface of NF-YB/NF-YC heterodimers (Hackenberg et al. 2012) and a DNA-binding domain (Fig. 3). The subunit interaction domain consists of 20 amino acids and alpha helix that is important for interaction with NF-YB and NF-YC subunits (Mantovani 1999). The DNA-binding domain of 21 amino acids is separated from the subunit interaction domains by a relatively conserved linker. Most mammalian and yeast functionally required amino acid residues (Maity and de Crombrugghe 1992; Xing et al. 1993) are present in the soybean NF-YA proteins. There are some alterations for alanine (A_4_ and A_16_) and leucine (L_11_) in soybean which can be replaced by hydrophobic residues (Maity and de Crombrugghe 1992) and arginine (R_9_) is commonly replaced by glycine and alanine which is usually seen in plant NF-YA (Siefers et al. 2009). Among soybean NF-Y proteins, only NF-YA subunits possess a nuclear localization signal (Kahle et al. 2005; Thon et al. 2010) with the presence of all three clusters of basic residues (Fig. 3). The N-terminus of NF-YAs is acidic and rich in glutamines and serine/threonine at N-termini, and loosely conserved and might function as an activation domain (Mantovani 1999).

**Fig. 4:**
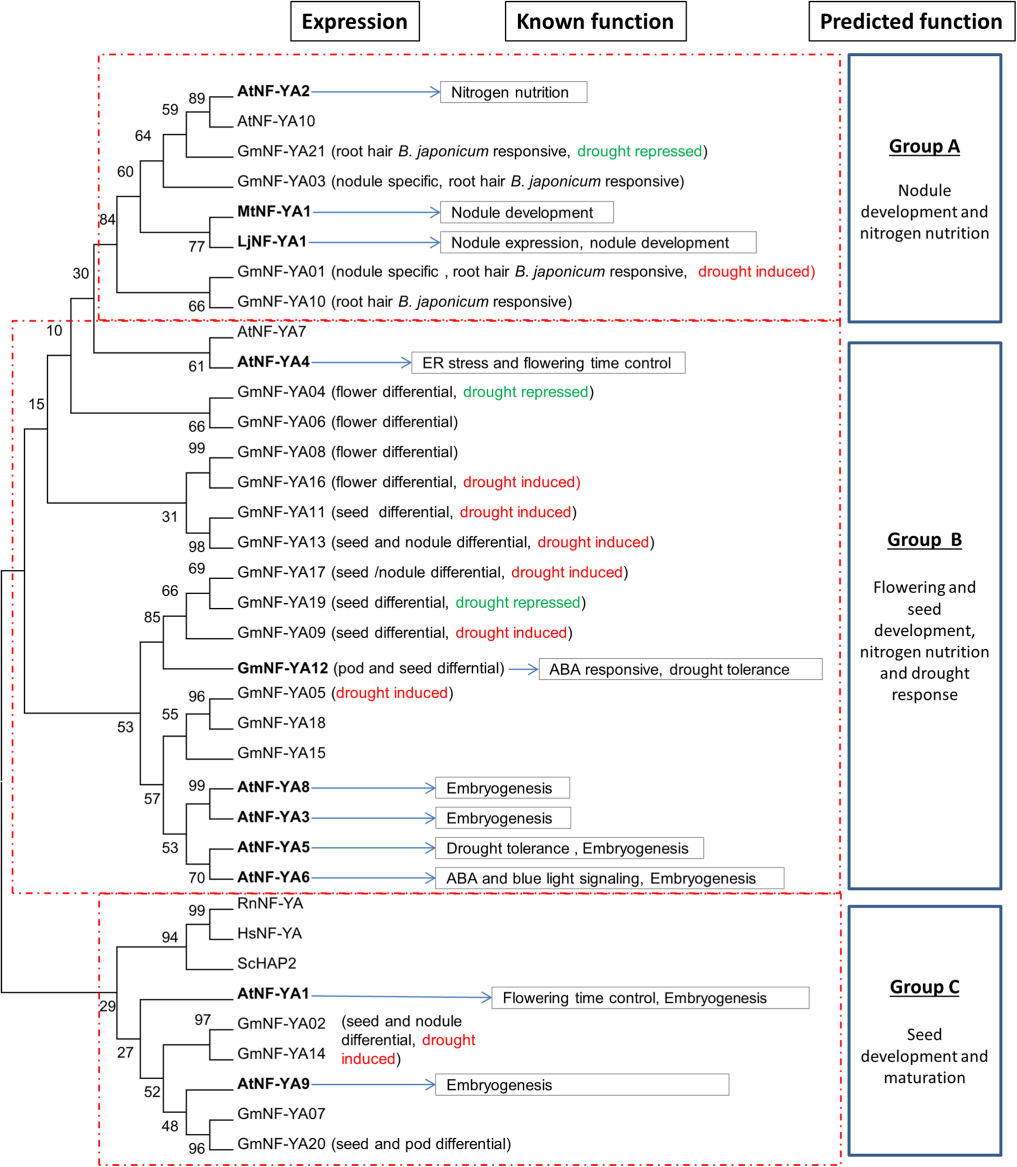
Phylogenetic tree of soybean NF-YA. The tree was constructed using MEGA5 software (see "Materials and methods") for the NF-YA domain sequences. *Bootstrap values* (from 1,000 replicates) were shown to represent the reliability of tree branches. The referred sequences are from *Arabidopsis* (At), *Medicago* (Mt), *L. japonicus* (Lj) human (Hs), mouse (Rn) and yeast (Sc). Function prediction for soybean genes were based on gene expression and homology to the referred characterized proteins which are written in *bold* for AtNF-YA1 (Wenkel et al. 2006; Mu et al. 2013), AtNF-YA2 (Zhao et al. 2011), AtNF-YA3 (Fornari et al. 2013), AtNF-YA4 (Wenkel et al. 2006; Liu and Howell 2010), AtNF-YA5 (Li et al. 2008; Mu et al. 2013), AtNF-YA6 (Fornari et al. 2013; Mu et al. 2013), AtNF-YA8 (Fornari et al. 2013), AtNF-YA9 (Mu et al. 2013), MtNF-YA1 (Combier et al. 2006), LjNF-YA4 (Soyano et al. 2013) and GmNF-YA12 (Ni et al. 2013)

A number of functional groups can be putatively assigned (Fig. 4). Group A consists of GmNF-YA01, GmNF-YA03 and GmNF-YA10 which are expressed highly in nodules and have sequences highly similar to the *M. truncatula* MtNF-YA1 (Combier et al. 2006), *Lotus japonicus* LjNF-YA1 (Soyano et al. 2013) and *Arabidopsis* AtNF-YA2 (Zhao et al. 2011), which are important in nodule development and nitrogen nutrition. These three nodule-specific genes are also induced strongly by inoculation with *B. japonicum* (Fig. 1). Group B contains the largest number of soybean GmNF-YA which have differential expression in flower and seeds and share sequence similarity with at least five functionally characterized proteins relating to ABA/dehydration signaling in seed development. Of these, GmNF-YA12 [known as GmNF-YA3 (Ni et al. 2013)] is induced by ABA, NaCl, PEG and cold treatments and controlled by miRNA169 to regulate drought tolerance in the *Arabidopsis* model (Ni et al. 2013). Genes in group B have diverse functions: AtNF-YA3 and AtNF-YA8 which regulate embryogenesis (Fornari et al. 2013) and AtNF-YA5 and AtNF-YA6 which regulate ABA and blue light signaling and drought tolerance (Warpeha et al. 2007; Li et al. 2008), and AtNF-YA4 which controls ER stress tolerance and flowering time (Wenkel et al. 2006; Liu and Howell 2010). In contrast, genes in group C might be particularly functioning in ABA-related seed development and maturation which are relevant to the functions of *Arabidopsis* AtNF-YA1 and AtNF-YA9 (Levesque-Lemay et al. 2003; Wenkel et al. 2006; Li et al. 2013; Mu et al. 2013). Sequence analysis predicted that 12 soybean GmNF-YA genes are targeted by miR169d and miR169e (TAG_5193213 and TAG_1196776, respectively). Expression miR169d was not associated with any of the NF-YA; however, expression of miRNA169e was negatively associated with GmNF-YA02, GmNF-YA12, GmNF-YA14 and GmNF-YA20 (data not shown). GmNF-YA12 has been shown to be cleaved by miR169e in a coexpression study in *Nicotiana benthamiana* and 5′ RACE assays (Ni et al. 2013).

#### NF-YB

**Fig. 5:**
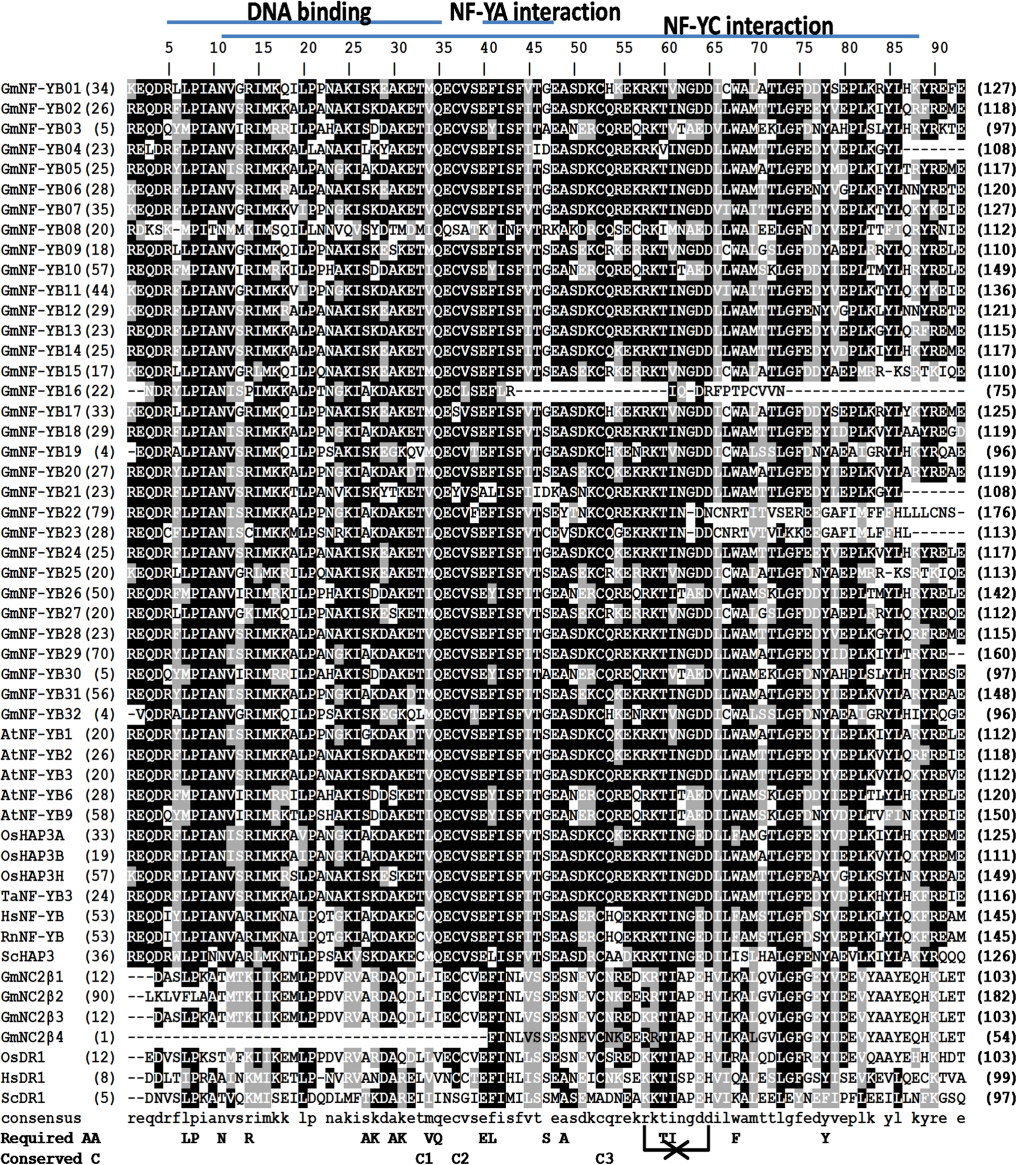
Alignment of soybean NF-YB domains. The referred sequences are from *Arabidopsis* (At), rice (Os), wheat (Ta), human (Hs), mouse (Rn) and yeast (Sc). *Numbers in parentheses* are the actual amino acid numbers of the start and end of NF-YB domain. Required amino acids (Required AA) are important for DNA binding and subunit interaction from *S*. *cerevisiae* (Xing et al. 1993) and rat (Maity and de Crombrugghe 1992). Conserved cysteine C1, C2, C3 of NF-YB and the putative H-bonds between arginine (R) and aspartate (D) were indicated in the *bottom line*. NC2β (or DR1) group which is homologous to NF-YB are included

The NF-YB subunits are characterized by the conserved NF-YA and NF-YC interaction domains and a DNA-binding domain (Fig. 5). Sequence structure of NF-YB is related to histone fold motif of H2B histone (Mantovani 1999). Histone has a conserved domain of 65-amino acid HFM with low sequence identity but high structural resemblance (Arents and Moudrianakis 1995). In *Arabidopsis* the combined surface of the NF-YB/NF-YC heterodimer is required for the interaction with NF-YA subunit and the translocation of the complex into nucleus (Hackenberg et al. 2012).

The residues required for the subunit interaction and DNA binding (Xing et al. 1993; Sinha et al. 1996) are highly conserved among GmNF-YB proteins except for relatively few alterations by similar functional residues (Fig. 5). Arginine (R_55_) and aspartate (D_65_) are suggested to have a crucial role in protein interaction due to possession of an important intramolecular hydrogen bond within the HFM (Romier et al. 2003; Hackenberg et al. 2012). These arginine–aspartate pairs are highly conserved through evolution in almost plants and animal species and are important in subunit interactions between NF-YB and NF-YC (Romier et al. 2003; Hackenberg et al. 2012). They are present in all soybean NF-YB proteins except GmNF-YB16 and GmNF-YB22 which have deletions in this domain and might not be able to form the NF-Y complex. Given the number of the GmNF-YB is almost three times while the numbers of GmNF-YA and GmNF-YC are about two times of corresponding genes in *Arabidopsis*, it is likely that the GmNF-YB proteins have gone through largest functional alteration.

Analysis of domain sequence of *Arabidopsis* NF-YB proteins reveals that the translocation of plant NF-Y complex into nucleus follows the piggyback transport model (Hackenberg et al. 2012) in which NF-YB and NF-YC heterodimer is formed first by head-to-tail annealing of their HFMs (Thon et al. 2010). A disulfide bond between two cysteines of NF-YB (C_33_ and C_37_) controls the heterodimerization of two subunits and further controls the spatial distribution of subunits and subcellular localization of the NF-Y complex upon a redox state. In soybean, all NF-YBs apparently lack the critical cysteine at position 33 and cannot form the disulfide bond (Fig. 5) which resembles the model of nuclear translocation of NF-Y complex in *Arabidopsis* (Hackenberg et al. 2012). Therefore, the redox-regulated assembly of NF-YB and NF-YC of human and *Aspergillus nidulans* is not present in the soybean NF-Y.

**Fig. 6:**
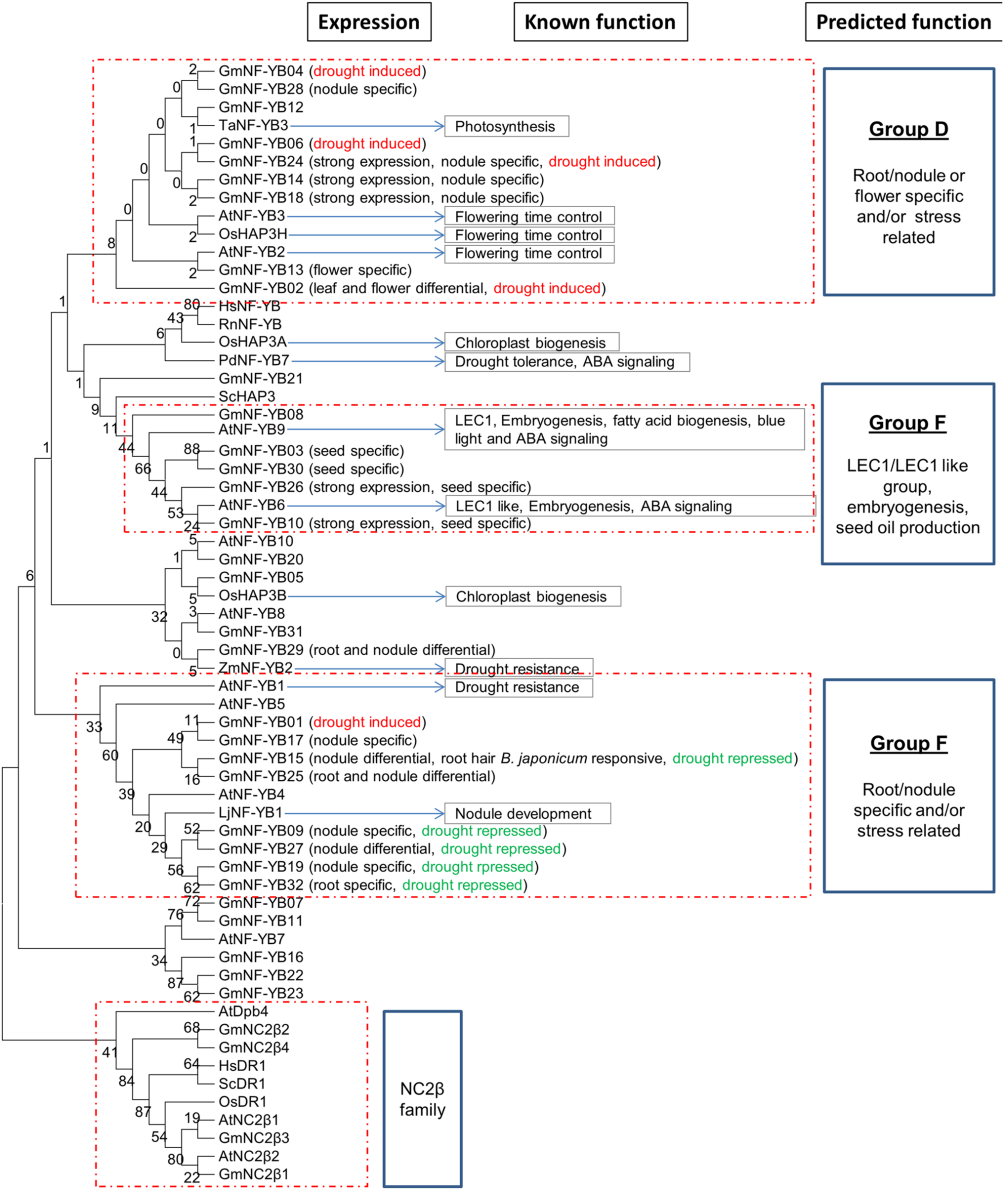
Phylogenetic tree of soybean NF-YB. Function prediction for soybean genes were based on gene expression and homology to the referred characterized proteins which are written in *bold* for AtNF-YB2 and AtNF-YB3 (Kumimoto et al. 2008), AtNF-YB6 (Lotan et al. 1998; Kwong et al. 2003; Lee et al. 2003; Tan et al. 2011), AtNF-YB9 (Kwong et al. 2003; Lee et al. 2003; Warpeha et al. 2007), OSHAP3 (Miyoshi et al. 2003), AtNF-YB1 and ZmNF-YB2 (Nelson et al. 2007) and LjNF-YB1 (Soyano et al. 2013)

NF-YB proteins can be putatively assigned to several functional groups (Fig. 6). Group D contains proteins that are closely related to the wheat TaNF-YB3 which regulates photosynthesis (Stephenson et al. 2011), *Arabidopsis* AtNF-YB2 and AtNF-YB3, and the rice OsHAP3S which controls flowering time control (Kumimoto et al. 2008; Wei et al. 2010). Surprisingly, many of the soybean genes are highly expressed in nodule suggesting that they may function in nodulations and nitrogen nutrition. Members of group E including GmNF-YB03, GmNF-YB10, GmNF-YB26 and GmNF-YB30 share homology to LEC1 and LEC1-like (AtNF-YB9 and AtNF-YB6). All these sequence have an aspartate at position 28 (D_28_) which is critical for LEC1 function and is not present in all other members of NF-YB proteins (Lee et al. 2003) indicating the unique function of this LEC1 group in embryogenesis and ABA signaling (Lotan et al. 1998; Kwong et al. 2003; Warpeha et al. 2007). LEC1 and LEC1-like orthologs have a conserved function in fatty acid biogenesis and seed oil production in *Brassica napus* (Tan et al. 2011; Mu et al. 2013) and maize (Shen et al. 2010). Group F composed a cluster of root/nodule differentially expressed NF-YB genes homologous to the *L. japonicus* protein LjNF-YB1, which together with LjNF-YA1 regulates division of cortical cells, an initial step in root nodule organogenesis (Soyano et al. 2013). Surprisingly, majority of nodule differential and rhizobium inducible genes in this group are drought repressed. Their close *Arabidopsis* homolog, *AtNF*-*YB1*, is an important gene regulating drought tolerance (Nelson et al. 2007).

Among three NF-Y subfamilies, only members of plant NF-YB have been identified to involve photosynthesis so far. Rice OsHAP3 genes regulate the expression of nuclear-encoded chloroplast-targeted genes and normal development of chloroplasts (Miyoshi et al. 2003). Down-regulation of OsHAP3A, OsHAP3B and OsHAP3C resulted in reduced chlorophyll and degenerated chloroplasts which failed to develop lamella and accumulate starch. These plants showed reduced expression of nuclear-encoded photosynthesis genes. In accordance with this, a wheat TaNF-YB3 was identified to co-express with photosynthetic genes and the transgenic plants showed increased leaf chlorophyll content and photosynthesis (Stephenson et al. 2011). Although close orthologs can be found in soybean, these genes do not belong to a certain phylogenetic tree branch (Fig. 6) making it unreliable to predict soybean NF-YB genes functioning in photosynthesis.

#### NF-YC

**Fig. 7:**
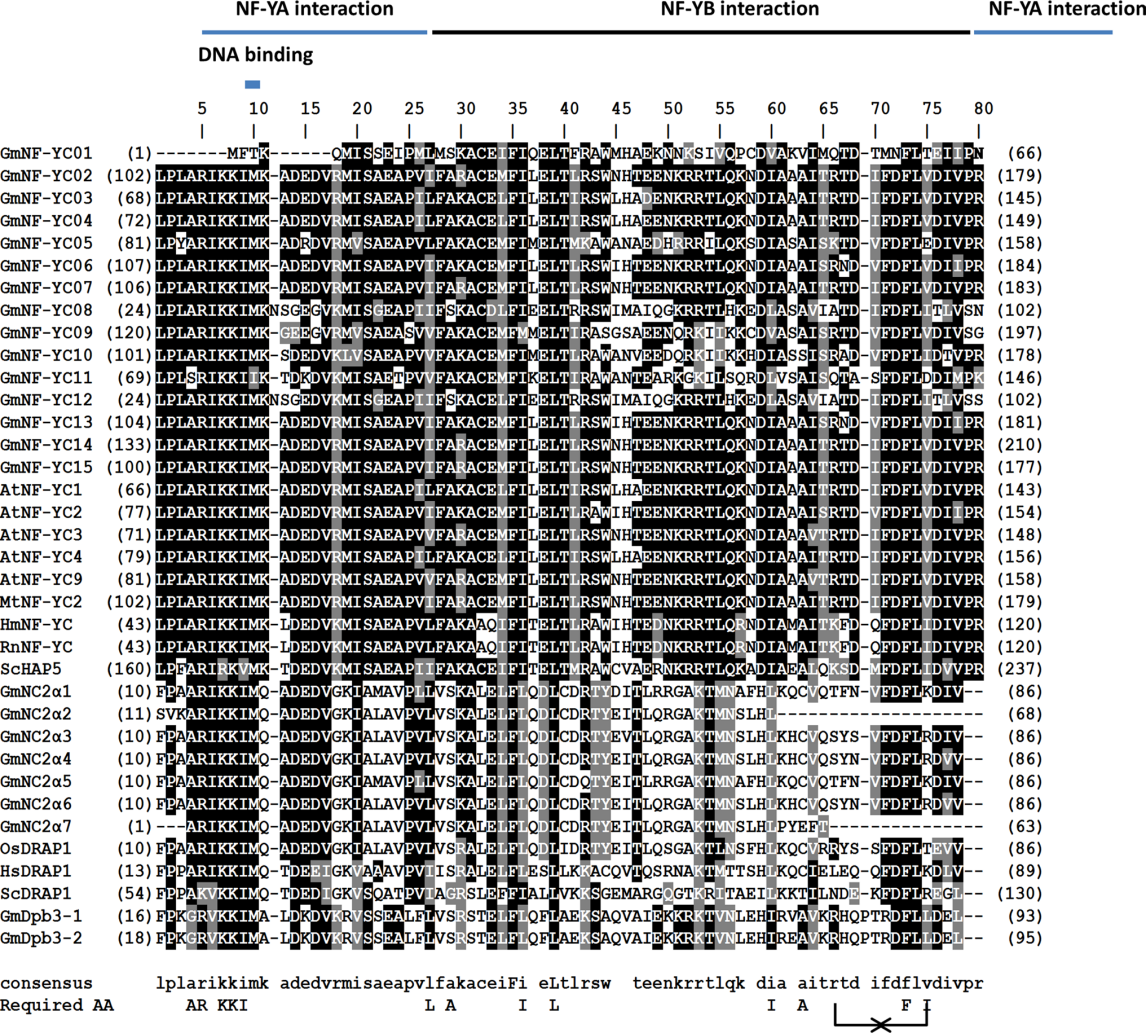
Alignment of soybean NF-YC domains. The referred sequences are from *Arabidopsis* (At), *Medicago* (Mt), human (Hs), mouse (Rn) and yeast (Sc). *Numbers in parentheses* are the actual amino acid numbers of the start and end of domain NF-YC. Required amino acids (Required AA) are important for DNA binding and subunit interaction from yeast (Xing et al. 1993) and rat (Maity and de Crombrugghe 1992). The putative H-bonds between arginine (R) and aspartate (D) were indicated in the bottom line. NC2α and Dpb (or DRAP1) groups which are homologous to NF-YC are included

Soybean genome has 15 genes encoding NF-YC subunits which have between 112 and 291 amino acids in length. GmNF-YC possesses of a highly conserved domain for NF-YA and NF-YB interaction (phylogenetic tree bootstrap values are substantially low, Fig. 7) and have structural and amino acid homology to H2A histone (Mantovani 1999). The majority of the required amino acids for the subunit interaction and DNA binding of NF-YC (Kim et al. 1996; Sinha et al. 1996) are highly conserved with minimal replacement of alternative residues of the same functional properties. The two amino acids arginine (R_52_) and aspartate (D_59_) necessary in stabilizing the structural conformations of NF-YB and NF-YC through the HFMs for mutual interaction between the NF-YB and NF-YC subunits (Hackenberg et al. 2012) are present in most soybean NF-YC, except for GmNF-YC11 (Fig. 7). GmNF-YC genes are expressed in various tissues except a small group of genes which are expressed specific in early seed development stages. Compared to NF-YA and NF-YB subfamily, fewer NF-YC genes showed response to drought treatment with only GmNF-YC15 is induced and GmNF-YC01, GmNF-YC07 and GmNF-YC12 are suppressed (Fig. 2).

**Fig. 8:**
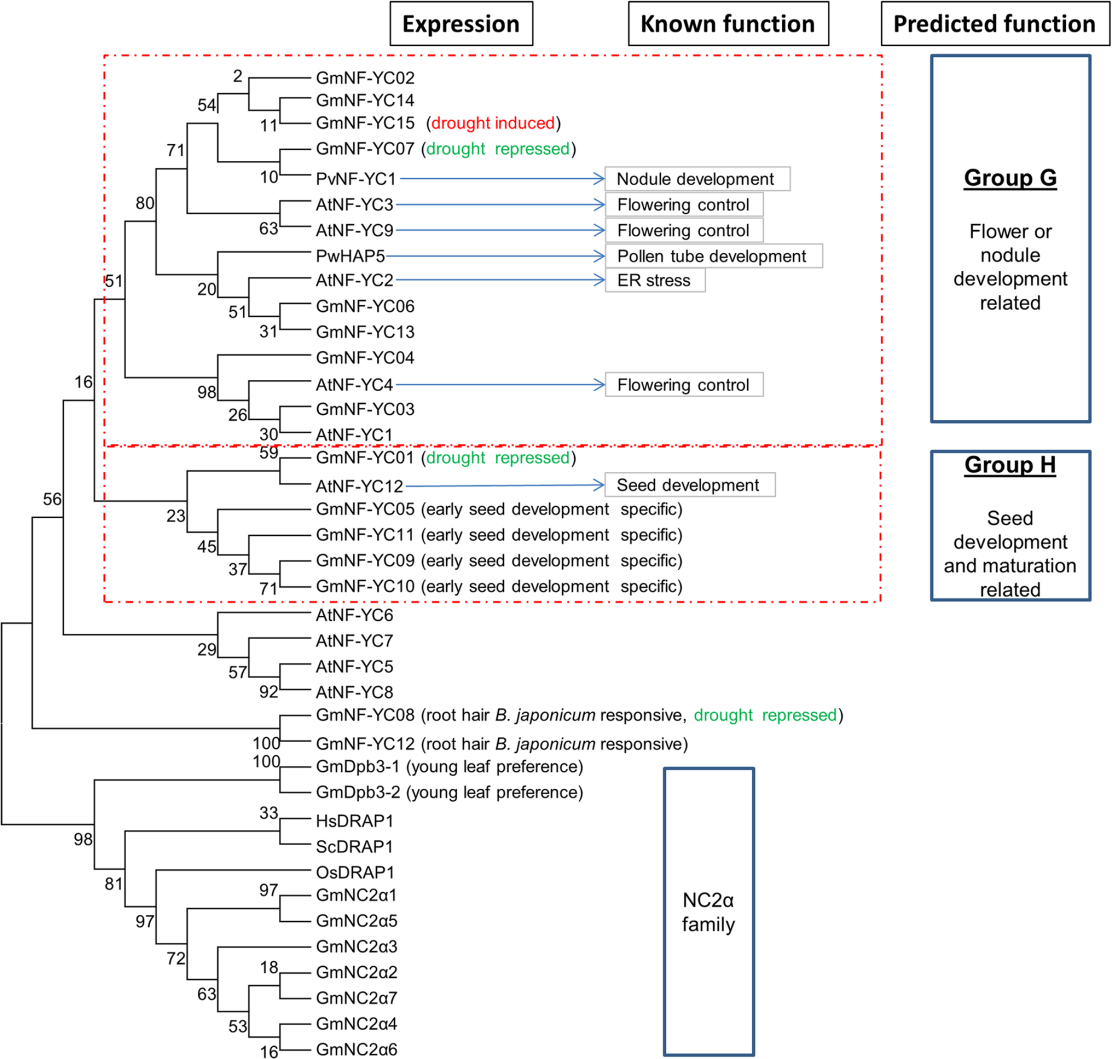
Phylogenetic tree of soybean NF-YC. Function prediction for soybean genes was based on gene expression and homology to the referred characterized proteins which are written in *bold* for AtNF-YC2 (Liu and Howell 2010), AtNF-YC3, AtNF-YC4 and AtNF-YC9 (Kumimoto et al. 2010), PvNF-YC1 (Zanetti et al. 2010) and PwHAP5 (Yu et al. 2011)

The sequences of NF-YC proteins are highly conserved (Fig. 8). The bootstrap supporting for most clades is quite low (generally <70 %); making the tree not necessarily accurate for lineage inferences. Although a crucial unit of NF-Y complex, less is known about the function of NF-YC proteins in plants. Limited work in *Arabidopsis* and *Medicago* showed that individual NF-YC is involved in nodule development, flowering control, ER stress response and ABA response in seed germination. Although the sequences are highly similar, the functions of previously characterized genes within tree branches appear too diverse (Fig. 8) to be used to assign a function within the NF-YC family. Group G includes genes with diverse functions relating to flower time control, stress adaptation and development (Ben-Naim et al. 2006; Wenkel et al. 2006). In *Arabidopsis*, NF-YC3, NF-YC4 and NF-YC9 are required for the CONSTANS-mediated, photoperiod-dependent regulation of the flowering locus FT (Kumimoto et al. 2010). The soybean members in group G also share homology with the *Arabidopsis* AtNF-YC2, which controls ER stress adaptation via unfolded protein response (Liu and Howell 2010), and the common bean (*Phaseolus vulgaris*) PvNF-YC1 which has been identified to positively regulate symbiotic interaction between the plant and the rhizobia (Zanetti et al. 2010). PvNF-YC1 activates cortical cell divisions and promotes nodule development in response to bacterial infection. Another close ortholog, *Picea wilsonii* PwHAP5, on the other hand, is involved in pollen tube development (Yu et al. 2011). In contrast, genes belonging to group H have protein sequences very conserved and possibly function in seed development due to specific expression in seeds and share strong homology to an *Arabidopsis* seed development-related gene AtNF-YC12. It is important to note that two soybean *GmNF*-*YC08* and *GmNF*-*YC12* are induced by the inoculation with *B. japonicum* indicating that these two genes might be specific to soybean response to the inoculation.

#### NC2

Our analysis also shows that the NC2α and Dpb groups share some homology to NF-YC proteins and NC2β shares homology to NF-YB (Petroni et al. 2012). Although there is sequence similarity, NF-YC cannot pair with NC2β and NF-YB cannot pair with NC2α (Zemzoumi et al. 1999). This binding preference might be explained by the difference in sequence structure, specifically the W44 of NF-YC at the end of helix alpha2 (Fig. 7), which is not present in NC2α. Additional to this difference, the hydrophobic cores organizing L1 and L2 regions are also different from that of NC2 (Romier et al. 2003). Although it is sharing some homology, all NC2 and Dpb3/Dpb4 genes are classified in a separate clade from phylogenetic tree analysis (Figs. 6, 8). In addition, NC2 and Dpb3/Dpb4 appear to have different functions such as NC2 associates with TATA box-binding protein to bind TATA box in the core promoters and the Dpb4/Dpb4 complex with DNA polymerase ε and the chromatin-remodeling complex CHRAC (Kamada et al. 2001; Hartlepp et al. 2005).

## Discussion

In the present report, we attempted to incorporate all the current annotations to explore the soybean NF-Y genes. Based on phylogenetics and up to date annotations, we identify 21 NF-YA, 32 NF-YB and 15 NF-YC and assign putative roles in plant growth and development, rhizobium-soybean symbiosis and drought adaptation for each subgroups. The updated clarification between NC2, Dpb3/4 and NF-Y (Petroni et al. 2012) allows accurate classification of NF-Y genes and updates databases and literature of previously characterized ones in plants including *A. thaliana*, *O. sativa*, *T. aestivum*, *Brachypodium distachyon*, *Selaginella moellendorffii* and *Brassica napus* (Stephenson et al. 2007; Thirumurugan et al. 2008; Siefers et al. 2009; Cao et al. 2011; Liang et al. 2013; Saha et al. 2013). Although there is high primary sequence similarity among these gene families, NC2 and Dpb3/4 groups are the outliners in the reported analyses from wheat, *Arabidopsis* and *B. distachyon* (Stephenson et al. 2007; Siefers et al. 2009; Cao et al. 2011). There is also a distinct functionality of NC2 groups: NC2 complex works as a global transcriptional repressor that targets TBP, thereby blocking formation of the transcription preinitiation complex (Kim et al. 1997). To this end, it is also necessary to update latest annotations for proper classification for the soybean transcription databases that are currently active (Guo et al. 2008; Mochida et al. 2010; Wang et al. 2010). With the recent reports describing the involvement of NF-Y in nodule and seed development, and nitrogen-fixing bacterium interactions (Combier et al. 2006; Libault et al. 2010; Severin et al. 2010; Soyano et al. 2013; Battaglia et al. 2014; Laloum et al. 2014; Ripodas et al. 2014; Singh et al. 2014) and our current transcriptional analysis under drought treatments, our analyses suggest possible roles of soybean NF-Y genes in seed and nodule development and drought responses.

### Plant fertility, embryogenesis and seed development

The involvement of NF-Y genes in developmental processes, particularly seed development and embryogenesis, has been identified in all three subfamilies (Petroni et al. 2012; Laloum et al. 2013). As expected, our transcript analysis identified several groups of soybean NF-YA, NF-YB and NF-YC which have highly specific or differential expression in soybean seeds and close relationship to the known genes. GmNF-FA subfamily appears to have more genes (8 out of total 16 GmNF-YA genes with available expression data) highly and differentially expressed in the seeds (Fig. 1). Their *Arabidopsis* orthologs, AtNF-YA1, AtNF-YA3, AtNF-YA5, AtNF-YA6, AtNF-YA8 and AtNF-YA9, have conserved and redundant function in male gametogenesis, embryogenesis and seed development (Fornari et al. 2013; Mu et al. 2013). Because the majority of the NF-YA genes specifically or differentially expressed in seeds are also induced by drought treatment (Fig. 1), they might be involved in the dehydration-response maturation process during seed development. Similar to the soybean seed development-related GmNF-YA, the soybean NF-YB (GmNF-YB03, GmNF-YB10, GmNF-YB26 and GmNF-YB30, Figs. 5, 6) are highly similar to the *Arabidopsis* AtLEC1 and L1L (Meinke 1992; West et al. 1994; Parcy et al. 1997; Lotan et al. 1998; Kwong et al. 2003; Junker et al. 2012). LEC1 and L1L are particularly different from other NF-YB proteins and appear to possess unique residues that are important to regulate hormonal and light signaling during embryogenesis (Lee et al. 2003; Junker et al. 2012). Indeed, their orthologs in other plant species showed the highly conserved function in regulation of the transition from embryo to adult status (Zhang et al. 2002; Yazawa et al. 2004; Alemanno et al. 2008; Mu et al. 2008; Schellenbaum et al. 2008; Shen et al. 2010; Cao et al. 2011; Tan et al. 2011). In addition, overexpression of LEC1, L1L and their orthologs resulted in seed oils via activation of various genes regulating fatty acid biosynthesis (Mu et al. 2008; Shen et al. 2010; Tan et al. 2011). Seed-specific expression is also seen in soybean GmNF-YC group for NF-YC05, NF-YC09, NF-YC10, NF-YC11. These genes share sequence similarity with the *Arabidopsis* AtNF-YC12, a flower differentially expressed gene (Siefers et al. 2009). During seed developmental stages, AtNF-YC12 is highly expressed in chalazal endosperm and chalazal seed coat of green mature seed (http://www.seedgenenetwork.net/). AtNF-YC12 interacts bilaterally with LEC1 and L1L in yeast—two hybrid assays (Hackenberg et al. 2012) indicating that they co-regulate seed development and embryogenesis. Therefore, it would be interesting to investigate the functions of soybean gene combinations in the control of embryogenesis and seed oil production in soybean.

Increasing evidences have showed that NF-Y genes are key regulators of flowering time control in plants. *Arabidopsis* AtNF-YB2 and AtNF-YB3 promote flowering in response to inductive long-day condition (Kumimoto et al. 2008) through regulation of expression of FLOWERING LOCUS T (FT), a key gene that controls vegetative to floral meristem conversion (Mathieu et al. 2007). AtNF-YB2 and AtNF-YB3 interact with three AtNF-YC (NF-YC3, NF-YC4 and NF-YC9), which physically interact with CONSTANS (CO), and are required for CO-mediated floral promotion to induce FT expression in the phloem tissue of long-day young leaves (Kumimoto et al. 2010). CO protein shares some homology to the *Arabidopsis* NF-YA subunits. Since overexpression of NF-YA1 and NF-YA4 caused delayed flowering, which may due to the impaired formation of NF-YB/NF-YC/CO (Wenkel et al. 2006). It is noted that the genes required for flowering time control often expressed in leaf vascular where CO genes are expressed (Kumimoto et al. 2010; Cao et al. 2011). Among the soybean NF-Y genes that are differentially expressed in young leaf, *GmNF*-*YB02*, *GmNF*-*YB13*, *GmNF*-*YC04*, *GmNF*-*YC06* and *GmNF*-*YC14* are particularly of interest for their role in regulation of flowering time in soybean due to close sequence relationship with *Arabidopsis* and rice flowering time control genes *AtNF*-*YB2*, *AtNF*-*YB3*, *OsHAP3H*, *AtNF*-*YC3* and *AtNF*-*YC9* (Miyoshi et al. 2003; Kumimoto et al. 2008, 2010).

### Nitrogen nutrition and nodulation

Nodulation is the result of a mutualistic interaction between the plant and symbiotic soil bacteria rhizobia, initiated by the infection of plant root hair cells by the symbiont. Initially, flavonoids secreted by the plant lead to the synthesis and secretion of a bacterial Nod factor (NF), which composes of chitooligosaccharide. Upon perception of NF, the symbiotic system activates various factors, of which, the nodule inception (NIN) and its downstream gene early nodulin (ERN) transcription factors are required for initiating nodulation-specific symbiotic processes (Madsen et al. 2010). In *L. japonicus*, NIN activates of the NF-Y subunit genes, *LjNF*-*YA1* and *LjNF*-*YB1* to induce cortical cell division, which is an initial step in root nodule organogenesis (Soyano et al. 2013). Similarly, in *M. truncatula*, the MtNF-YA1 (MtHAP2-1) gene which shares homology to LjNF-YA1 is important in nodule development and plant–bacterial symbiotic interaction (Combier et al. 2006; Laporte et al. 2013; Laloum et al. 2014). It is highly expressed in mature nodules, first induced at the onset of symbiotic development during preparation for, and initiation and progression of, symbiotic infection. MtNF-YA1 controls rhizobial infection progression by regulating the formation and the wall of infection thread. In *M. truncatula*, MtNF-YA1 and MtNF-YA2 have a redundant function in nodulation development via ERN1 activation (Laloum et al. 2014). Our analysis in soybean shows that the *M. truncatula* MtNF-YA1 and *L. japonicus* LjNF-YA1 share strong homology to several soybean NF-YA genes (group A: *GmNF*-*YA01*, *GmNF*-*YA03*, *GmNF*-*YA10* and *GmNF*-*YA21*). These genes are expressed specifically in nodules and induced by *B. japonicum* (Fig. 4), indicating a function in the symbiotic interaction and nodule development. We also found that genes in group F of GmNF-YB subfamily are specifically expressed in nodule and share a high sequence homology to *L. japonicus* LjNF-YB1 (Fig. 6), which regulates nodule development in interaction with LjNF-YA1, downstream of NIN (Soyano et al. 2013). Within this group, GmNY-YB15 is of particular interest for its nodule differential and rhizobium induced, suggesting a role in initial root nodule organogenesis. Members of subfamily NF-YC, on the other hand, are least known for the involvement in the nodulation development process. Among 15 soybean NF-YC genes, only GmNF-YC08 is induced by *B. japonicum* treatments (Supplementary Figure S2), suggesting an involvement in initial process of nodule development. Up to date, the *Phaseolus vulgaris* PvNF-YC1 is the first and the only NF-YC known to be required, and plays a key role in the nodule organogenesis and rhizobium infection (Zanetti et al. 2010; Mazziotta et al. 2013). Overexpression of *PvNF*-*YC1* improves nodulation efficiency on the less-efficient rhizobium strains, possibly through activation of the genes in G2/M transition. NF-YC1 could interact physically with SIN1, a GRAS-type transcription factor family which is required for efficient nodule development.

### Stress response and adaptation

Increasing evidences have shown that individual NF-Y subunits, particularly NF-YA and NF-YB, are important to regulate drought and ER stress tolerance in both dicots and monocots with various mechanisms including tolerance to reduced water loss and low tissue water potential (Nelson et al. 2007; Li et al. 2008; Liu and Howell 2010; Han et al. 2013; Ni et al. 2013). The present investigation identified a large number of soybean NF-YA and NF-YB genes responsive to drought treatments and possibly involved in drought adaptation (Figs. 1, 2). Soybean NF-YA family appears to have more genes induced by drought (7 of 21 genes). Most these genes belong to the groups that have differential expression in the seeds, suggesting that they are involved in maturation and dehydration signaling. Three soybean genes: GmNF-YA09, GmNF-YA17 and GmNF-YA19 have sequence homology to the drought tolerance GmNF-YA12 [or GmNF-YA3 (Ni et al. 2013)], AtNF-YA5 and AtNF-YA6 proteins (Li et al. 2008; Fornari et al. 2013; Mu et al. 2013). *GmNF*-*YA12* was induced by abscisic acid (ABA) and abiotic stresses, such as NaCl and cold, and has a role in drought tolerance by reduced leaf water loss via regulation of the ABA signaling pathway. *AtNF*-*YA5* is induced by ABA, NaCl, cold and PEG treatment, and control water loss in *Arabidopsis* (Li et al. 2008). AtNF-YA5 also has a redundant role with AtNF-YA1, AtNF-YA6 and AtNF-YA9 which are downstream of LEC1 and regulate of embryogenesis and seed development in *Arabidopsis* (Mu et al. 2013). Most of drought-inducible soybean genes (5/7 genes) in this group are predicted to be targets of microRNA169 (data not shown), which is known to cleave GmNF-YA12 and AtNF-YA5 in response to drought stress (Li et al. 2008; Ni et al. 2013), indicating a conserved function in seed development and drought-response regulation.

Our phylogenetic analysis shows that two clades of soybean homology to the drought-tolerant *Arabidopsis* AtNF-YB1 and maize ZmNF-YB2 (Nelson et al. 2007). AtNF-YB1 and ZmNF-YB2 function in drought tolerance by maintaining leaf water potential, stomatal conductance and photosynthesis (Nelson et al. 2007). Surprisingly, most of the genes sharing homology to AtNF-YB1 (group F) are repressed by drought treatment. The majority of these genes are in fact specific/differential to nodule. Therefore, it is unlikely that they are involved in drought tolerance in soybean. GmNF-YB01, the only homolog of drought-tolerant genes AtNF-YB1 and ZmNF-YB2, is expressed ubiquitously in various tissues and induced by drought (Fig. 6) suggesting that it is potentially involved in drought tolerance.

We found that the majority of genes that are highly specific to nodules are downregulated by drought stress. Soybean nodulation and nodule activity are strongly reduced under the stress (Sinclair et al. 2007) suggesting that NF-Y transcription factors are important in the regulation of nodule activity. Further characterization of these transcription factors will be useful in understanding of molecular regulation of nodulation and improvement of nitrogen assimilation during drought stress, as maintenance of nitrogen fixation is highly associated with maintenance of soybean productivity under the stress (Sinclair et al. 2007).

## Electronic supplementary material

Below is the link to the electronic supplementary material.

Supplementary material 1 (DOCX 71 kb)

Supplementary material 2 (DOCX 307 kb)

Supplementary material 3 (DOCX 24 kb)

Supplementary material 4 (DOCX 22 kb)

Supplementary material 5 (DOCX 21 kb)

## Abbreviations

ABA: Abscisic acid
DAF: Days after flowering
ER: Endoplasmic reticulum
ERN: Early nodulins
FC: Fold change
HFM: Histone fold motif
HMM: Hidden Markov Model
NF-Y: Nuclear factor Y
NIN: Nodule inception
qRT-PCR: Quantitative real-time PCR
RPKM: Reads per kilobase per million
TF: Transcription factor
