# Supplementary material for: Genome-wide expression analysis of soybean NF-Y genes reveals potential function in development and drought response
Source: Mol Genet Genomics. 2014 Dec 27;290(3):1095–115. doi: 10.1007/s00438-014-0978-2 (PMC4435856; doi:10.1007/s00438-014-0978-2)
Supplement: Supplementary file 2 — Supplementary material 2 (DOCX 307 kb) [file 438_2014_978_MOESM2_ESM.docx]

Supplementary Fig. S2 Expression of soybean NF-Y genes in response to *B. japonicum* treatments in root hair. Data were extracted from the soybean database, SOYKB (<http://soykb.org/data_sources.php>) with original data from soybean gene expression atlas ([Libault, Farmer et al. 2010](#_ENREF_1)). Root hairs were collected from 12, 24, and 48-hour *B. japonicum* inoculated roots and their corresponding un-inoculated controls. The color bar represents number of reads per kilobase per million (RPKM).

Genome-Wide Expression Analysis of Soybean NF-Y Genes Reveals Potential Function in Development and Drought Response

Truyen N Quach^1,2 §^, Hanh TM Nguyen^1,3 §^, Babu Valliyodan^1^, Trupti Joshi^4^, Dong Xu^4^, Henry T. Nguyen^1*^

^1^Division of Plant Sciences, National Center for Soybean Biotechnology, University of Missouri, Columbia, MO, USA

^2^Current address: Field Crop Research Institute, Vietnam Academy of Agricultural Sciences, Hanoi, Vietnam

^3^Current address: The Center for Plant Science Innovation, University of Nebraska, Lincoln, NE, USA

^4^Department of Computer Science, Christopher S. Bond Life Sciences Center, National Center for Soybean Biotechnology and Informatics Institute, University of Missouri, Columbia, MO, USA

^§^These authors contribute equally to the research

^*^Corresponding author:

Henry T. Nguyen

National Center for Soybean Biotechnology and Division of Plant Sciences, University of Missouri, Columbia, Missouri 65211, USA.

Tel: 573-882-5494

Fax: 573-882-1469

E-mail: [nguyenhenry@missouri.edu](mailto:nguyenhenry@missouri.edu)

Libault, M., A. Farmer, et al. (2010). "Complete transcriptome of the soybean root hair cell, a single-cell model, and its alteration in response to Bradyrhizobium japonicum infection." Plant Physiol **152**(2): 541-552.
