# Supplementary material for: Genome-wide expression analysis of soybean NF-Y genes reveals potential function in development and drought response
Source: Mol Genet Genomics. 2014 Dec 27;290(3):1095–115. doi: 10.1007/s00438-014-0978-2 (PMC4435856; doi:10.1007/s00438-014-0978-2)
Supplement: Supplementary file 3 — Supplementary material 3 (DOCX 24 kb) [file 438_2014_978_MOESM3_ESM.docx]

Supplementary Table S1 NF-Y, NC2 and Dpb genes in soybean and their orthologs in other plant species. The characterized proteins having highest homology to the soybean proteins in alignment analyses are included for reference. The referred sequences are from Arabidopsis (At), rice (Os), Medicago (Mt), Maize (Zm), and Wheat (Ta).

Genome-Wide Expression Analysis of Soybean NF-Y Genes Reveals Potential Function in Development and Drought Response

Truyen N Quach^1,2 §^, Hanh TM Nguyen^1,3 §^, Babu Valliyodan^1^, Trupti Joshi^4^, Dong Xu^4^, Henry T. Nguyen^1*^

^1^Division of Plant Sciences, National Center for Soybean Biotechnology, University of Missouri, Columbia, MO, USA

^2^Current address: Field Crop Research Institute, Vietnam Academy of Agricultural Sciences, Hanoi, Vietnam

^3^Current address: The Center for Plant Science Innovation, University of Nebraska, Lincoln, NE, USA

^4^Department of Computer Science, Christopher S. Bond Life Sciences Center, National Center for Soybean Biotechnology and Informatics Institute, University of Missouri, Columbia, MO, USA

^§^These authors contribute equally to the research

^*^Corresponding author:

Henry T. Nguyen

National Center for Soybean Biotechnology and Division of Plant Sciences, University of Missouri, Columbia, Missouri 65211, USA.

Tel: 573-882-5494

Fax: 573-882-1469

E-mail: [nguyenhenry@missouri.edu](mailto:nguyenhenry@missouri.edu)

| **Soybean gene** | **Primary Phytozome ID** | **Characterized proteins** | | **Putative Arabidopsis orthologs** | | |
| --- | --- | --- | --- | --- | --- | --- |
|  |  | **Gene** | **P-value** | **Locus** | **Gene** | **P-value** |
| GmNC2β1 | Glyma05g07750.1 | OsDR1 | 6.00E-61 | AT5G23090 | At-NC2β2 | 1.00E-71 |
| GmNC2β2 | Glyma06g23234.1 | OsDR1 | 2.00E-51 | AT5G23090 | At-NC2β2 | 1.00E-61 |
| GmNC2β3 | Glyma17g13260.1 | OsDR1 | 1.00E-60 | AT5G23090 | At-NC2β2 | 5.00E-72 |
| GmNC2β4 | Glyma18g22896.1 | OsDR1 | 2.00E-33 | AT5G08190 | At-NC2β1 | 2.00E-42 |
| GmNC2α1 | Glyma02g44500.2 | OsDRAP1 | 2.00E-54 | AT3G12480 | At-NC2α | 1.00E-69 |
| GmNC2α2 | Glyma06g04161.1 | OsDRAP1 | 4.00E-24 | AT3G12480 | At-NC2α | 2.00E-25 |
| GmNC2α3 | Glyma06g46850.1 | OsDRAP1 | 5.00E-56 | AT3G12480 | At-NC2α | 9.00E-83 |
| GmNC2α4 | Glyma13g25860.1 | OsDRAP1 | 8.00E-58 | AT3G12480 | At-NC2α | 2.00E-81 |
| GmNC2α5 | Glyma14g04321.1 | OsDRAP1 | 2.00E-55 | AT3G12480 | At-NC2α | 8.00E-70 |
| GmNC2α6 | Glyma15g36170.1 | OsDRAP1 | 2.00E-55 | AT3G12480 | At-NC2α | 2.00E-78 |
| GmNC2α7 | Glyma19g41280.1 | OsDRAP1 | 4.00E-24 | AT3G12480 | At-NC2α | 2.00E-25 |
| GmDpb3-1 | Glyma11g37130.1 | OsDRAP1 | 2.00E-11 | AT5G43250 | At-Dpb3-1 | 2.00E-38 |
| GmDpb3-2 | Glyma18g01040.1 | OsDRAP1 | 5.00E-12 | AT5G43250 | At-Dpb3-1 | 2.00E-39 |
| GmNF-YA01 | Glyma02g35190.4 | AtNF-YA10 | 3.00E-49 | AT5G06510 | At-NF-YA10 | 8.00E-53 |
| GmNF-YA02 | Glyma02g47380.5 | AtNF-YA9 | 2.00E-55 | AT3G20910 | At-NF-YA9 | 2.00E-55 |
| GmNF-YA03 | Glyma03g36140.5 | AtNF-YA10 | 6.00E-50 | AT5G06510 | At-NF-YA10 | 5.00E-53 |
| GmNF-YA04 | Glyma05g29970.1 | AtNF-YA4 | 1.00E-48 | AT1G30500 | At-NF-YA7 | 3.00E-55 |
| GmNF-YA05 | Glyma07g04050.9 | AtNF-YA3 | 7.00E-65 | AT1G72830 | At-NF-YA3 | 3.00E-66 |
| GmNF-YA06 | Glyma08g13090.1 | AtNF-YA4 | 5.00E-51 | AT1G30500 | At-NF-YA7 | 2.00E-58 |
| GmNF-YA07 | Glyma08g45030.1 | AtNF-YA1 | 3.00E-75 | AT5G12840 | At-NF-YA1 | 3.00E-75 |
| GmNF-YA08 | Glyma09g02770.2 | AtNF-YA4 | 5.00E-52 | AT1G30500 | At-NF-YA7 | 4.00E-56 |
| GmNF-YA09 | Glyma09g07960.4 | AtNF-YA3 | 5.00E-34 | AT1G72830 | At-NF-YA3 | 2.00E-35 |
| GmNF-YA10 | Glyma10g10240.2 | AtNF-YA10 | 4.00E-49 | AT5G06510 | At-NF-YA10 | 3.00E-54 |
| GmNF-YA11 | Glyma12g36540.6 | AtNF-YA9 | 2.00E-39 | AT3G20910 | At-NF-YA9 | 2.00E-39 |
| GmNF-YA12 | Glyma13g16770.1 | AtNF-YA3 | 3.00E-35 | AT1G72830 | At-NF-YA3 | 1.00E-36 |
| GmNF-YA13 | Glyma13g27230.4 | AtNF-YA9 | 1.00E-40 | AT3G20910 | At-NF-YA9 | 1.00E-40 |
| GmNF-YA14 | Glyma14g01360.1 | AtNF-YA9 | 2.00E-55 | AT3G20910 | At-NF-YA9 | 2.00E-55 |
| GmNF-YA15 | Glyma15g03175.1 | AtNF-YA3 | 4.00E-37 | AT1G72830 | At-NF-YA3 | 2.00E-38 |
| GmNF-YA16 | Glyma15g13660.2 | AtNF-YA4 | 3.00E-51 | AT1G30500 | At-NF-YA7 | 4.00E-54 |
| GmNF-YA17 | Glyma15g18970.1 | AtNF-YA3 | 1.00E-33 | AT1G72830 | At-NF-YA3 | 6.00E-35 |
| GmNF-YA18 | Glyma16g00711.1 | AtNF-YA3 | 8.00E-51 | AT1G72830 | At-NF-YA3 | 3.00E-52 |
| GmNF-YA19 | Glyma17g05920.1 | AtNF-YA3 | 3.00E-33 | AT1G72830 | At-NF-YA3 | 1.00E-34 |
| GmNF-YA20 | Glyma18g07890.2 | AtNF-YA1 | 5.00E-77 | AT5G12840 | At-NF-YA1 | 5.00E-77 |
| GmNF-YA21 | Glyma19g38800.2 | AtNF-YA10 | 3.00E-52 | AT5G06510 | At-NF-YA10 | 5.00E-55 |
| GmNF-YB01 | Glyma02g17310.1 | TaNF-YB3 | 2.00E-44 | AT2G47810 | At-NF-YB5 | 3.00E-49 |
| GmNF-YB02 | Glyma02g46970.1 | AtNF-YB2 | 1.00E-59 | AT5G47640 | At-NF-YB2 | 1.00E-59 |
| GmNF-YB03 | Glyma03g18670.2 | AtNF-YB6 | 1.00E-47 | AT5G47670 | At-NF-YB6 | 2.00E-47 |
| GmNF-YB04 | Glyma03g22721.1 | TaNF-YB3 | 1.00E-45 | AT4G14540 | At-NF-YB3 | 2.00E-45 |
| GmNF-YB05 | Glyma03g33490.1 | ZmNF-YB2 | 2.00E-59 | AT3G53340 | At-NF-YB10 | 8.00E-72 |
| GmNF-YB06 | Glyma05g31681.1 | OsHAP3H | 2.00E-51 | AT2G13570 | At-NF-YB7 | 1.00E-49 |
| GmNF-YB07 | Glyma05g32680.1 | AtNF-YB3 | 4.00E-48 | AT2G13570 | At-NF-YB7 | 1.00E-62 |
| GmNF-YB08 | Glyma07g29695.1 | AtNF-YB6 | 3.00E-25 | AT1G21970 | At-NF-YB9 | 3.00E-25 |
| GmNF-YB09 | Glyma07g37840.2 | AtNF-YB2 | 7.00E-42 | AT2G47810 | At-NF-YB5 | 1.00E-43 |
| GmNF-YB10 | Glyma07g39820.1 | AtNF-YB6 | 2.00E-56 | AT5G47670 | At-NF-YB6 | 1.00E-61 |
| GmNF-YB11 | Glyma08g00330.2 | AtNF-YB3 | 1.00E-49 | AT2G13570 | At-NF-YB7 | 5.00E-64 |
| GmNF-YB12 | Glyma08g14931.1 | AtNF-YB3 | 1.00E-49 | AT4G14540 | At-NF-YB3 | 1.00E-49 |
| GmNF-YB13 | Glyma08g44140.2 | TaNF-YB3 | 3.00E-64 | AT5G47640 | At-NF-YB2 | 1.00E-59 |
| GmNF-YB14 | Glyma09g01650.1 | TaNF-YB3 | 8.00E-61 | AT4G14540 | At-NF-YB3 | 1.00E-59 |
| GmNF-YB15 | Glyma09g05150.2 | AtNF-YB2 | 3.00E-37 | AT2G47810 | At-NF-YB5 | 1.00E-39 |
| GmNF-YB16 | Glyma09g28671.1 | AtNF-YB1 | 2.00E-19 | AT2G38880 | At-NF-YB1 | 2.00E-19 |
| GmNF-YB17 | Glyma10g02480.1 | AtNF-YB2 | 1.00E-42 | AT2G47810 | At-NF-YB5 | 3.00E-47 |
| GmNF-YB18 | Glyma10g05606.1 | ZmNF-YB2 | 2.00E-57 | AT3G53340 | At-NF-YB10 | 3.00E-63 |
| GmNF-YB19 | Glyma10g29440.2 | AtNF-YB3 | 4.00E-36 | AT1G09030 | At-NF-YB4 | 2.00E-43 |
| GmNF-YB20 | Glyma10g33550.2 | AtNF-YB1 | 9.00E-60 | AT2G37060 | At-NF-YB8 | 2.00E-60 |
| GmNF-YB21 | Glyma11g18190.1 | AtNF-YB3 | 6.00E-42 | AT4G14540 | At-NF-YB3 | 6.00E-42 |
| GmNF-YB22 | Glyma11g29866.1 | ZmNF-YB2 | 1.00E-29 | AT3G53340 | At-NF-YB10 | 1.00E-32 |
| GmNF-YB23 | Glyma13g10690.2 | ZmNF-YB2 | 8.00E-28 | AT3G53340 | At-NF-YB10 | 7.00E-29 |
| GmNF-YB24 | Glyma15g12570.1 | AtNF-YB3 | 9.00E-59 | AT4G14540 | At-NF-YB3 | 9.00E-59 |
| GmNF-YB25 | Glyma15g16460.2 | AtNF-YB1 | 4.00E-38 | AT2G47810 | At-NF-YB5 | 9.00E-40 |
| GmNF-YB26 | Glyma17g00950.1 | AtNF-YB6 | 7.00E-57 | AT5G47670 | At-NF-YB6 | 5.00E-62 |
| GmNF-YB27 | Glyma17g02810.1 | AtNF-YB1 | 4.00E-41 | AT2G47810 | At-NF-YB5 | 6.00E-44 |
| GmNF-YB28 | Glyma18g08620.1 | TaNF-YB3 | 2.00E-63 | AT5G47640 | At-NF-YB2 | 2.00E-59 |
| GmNF-YB29 | Glyma19g36220.2 | ZmNF-YB2 | 4.00E-58 | AT3G53340 | At-NF-YB10 | 3.00E-64 |
| GmNF-YB30 | Glyma20g00240.1 | AtNF-YB6 | 3.00E-48 | AT5G47670 | At-NF-YB6 | 3.00E-48 |
| GmNF-YB31 | Glyma20g34050.2 | AtNF-YB1 | 2.00E-58 | AT2G37060 | At-NF-YB8 | 2.00E-60 |
| GmNF-YB32 | Glyma20g37870.2 | TaNF-YB3 | 2.00E-34 | AT1G09030 | At-NF-YB4 | 3.00E-43 |
| GmNF-YC01 | Glyma02g09867.1 | AtNF-YC1 | 4.00E-16 | AT3G48590 | At-NF-YC1 | 4.00E-16 |
| GmNF-YC02 | Glyma03g39911.1 | AtNF-YC9 | 2.00E-75 | AT1G08970 | At-NF-YC9 | 2.00E-75 |
| GmNF-YC03 | Glyma04g37291.1 | AtNF-YC1 | 6.00E-94 | AT3G48590 | At-NF-YC1 | 6.00E-94 |
| GmNF-YC04 | Glyma06g17780.1 | AtNF-YC1 | 9.00E-96 | AT3G48590 | At-NF-YC1 | 9.00E-96 |
| GmNF-YC05 | Glyma08g15700.1 | AtNF-YC9 | 3.00E-46 | AT1G08970 | At-NF-YC9 | 3.00E-46 |
| GmNF-YC06 | Glyma08g17630.1 | MtNF-YC2 | 2.00E-78 | AT1G56170 | At-NF-YC2 | 1.00E-74 |
| GmNF-YC07 | Glyma10g29691.1 | AtNF-YC9 | 7.00E-75 | AT1G08970 | At-NF-YC9 | 7.00E-75 |
| GmNF-YC08 | Glyma12g34510.1 | AtNF-YC1 | 2.00E-25 | AT3G48590 | At-NF-YC1 | 2.00E-25 |
| GmNF-YC09 | Glyma13g27770.2 | MtNF-YC2 | 7.00E-46 | AT1G54830 | At-NF-YC3 | 5.00E-45 |
| GmNF-YC10 | Glyma13g27780.2 | MtNF-YC2 | 3.00E-51 | AT1G08970 | At-NF-YC9 | 2.00E-47 |
| GmNF-YC11 | Glyma13g27790.1 | MtNF-YC2 | 2.00E-35 | AT1G54830 | At-NF-YC3 | 5.00E-35 |
| GmNF-YC12 | Glyma13g35980.1 | AtNF-YC1 | 9.00E-27 | AT3G48590 | At-NF-YC1 | 9.00E-27 |
| GmNF-YC13 | Glyma15g41486.1 | MtNF-YC2 | 1.00E-77 | AT1G56170 | At-NF-YC2 | 2.00E-73 |
| GmNF-YC14 | Glyma19g42460.1 | AtNF-YC9 | 6.00E-79 | AT1G08970 | At-NF-YC9 | 6.00E-79 |
| GmNF-YC15 | Glyma20g37620.1 | AtNF-YC9 | 1.00E-76 | AT1G08970 | At-NF-YC9 | 1.00E-76 |
