# Supplementary material for: Genome-wide expression analysis of soybean NF-Y genes reveals potential function in development and drought response
Source: Mol Genet Genomics. 2014 Dec 27;290(3):1095–115. doi: 10.1007/s00438-014-0978-2 (PMC4435856; doi:10.1007/s00438-014-0978-2)
Supplement: Supplementary file 4 — Supplementary material 4 (DOCX 22 kb) [file 438_2014_978_MOESM4_ESM.docx]

Supplementary Table S2 Primers for quantitative real time PCR of soybean NF-Y genes.

Genome-Wide Expression Analysis of Soybean NF-Y Genes Reveals Potential Function in Development and Drought Response

Truyen N Quach^1,2 §^, Hanh TM Nguyen^1,3 §^, Babu Valliyodan^1^, Trupti Joshi^4^, Dong Xu^4^, Henry T. Nguyen^1*^

^1^Division of Plant Sciences, National Center for Soybean Biotechnology, University of Missouri, Columbia, MO, USA

^2^Current address: Field Crop Research Institute, Vietnam Academy of Agricultural Sciences, Hanoi, Vietnam

^3^Current address: The Center for Plant Science Innovation, University of Nebraska, Lincoln, NE, USA

^4^Department of Computer Science, Christopher S. Bond Life Sciences Center, National Center for Soybean Biotechnology and Informatics Institute, University of Missouri, Columbia, MO, USA

^§^These authors contribute equally to the research

^*^Corresponding author:

Henry T. Nguyen

National Center for Soybean Biotechnology and Division of Plant Sciences, University of Missouri, Columbia, Missouri 65211, USA.

Tel: 573-882-5494

Fax: 573-882-1469

E-mail: [nguyenhenry@missouri.edu](mailto:nguyenhenry@missouri.edu)

| **Gene ID** | **Current gene name** | **Forward primer (5'-3')** | **Reverse primers (5'-3')** | **qRT-PCR product** |
| --- | --- | --- | --- | --- |
| Glyma02g44500.2 | GmNC2α1 | CTTCCCCTTTATAAAAACCAAAC | CTTGCATAATTTTCTTAATCCTAG | Yes |
| Glyma06g04161.1 | GmNC2α2 | CGAAAACACCTAACCCTAATATTG | CGAATTTTTCAGTAGAAAATGG | Yes |
| Glyma06g46850.1 | GmNC2α3 | AGAGGCCGAGGTAGAGGAAG | GCAGAATAGAGGTGCAAGGC | Yes |
| Glyma13g25860.1 | GmNC2α4 | TGTTGAAAGCACTGATTCGC | CATGCTTGCTCTCTGTTGGA | Yes |
| Glyma14g04321.1 | GmNC2α5 | TATGCAAGCTGACGAGGATG | ATTCATAGTCTTTGCCCCCC | Yes |
| Glyma15g36170.1 | GmNC2α6 | CACTGGCTGTGCCTGTTTTA | TCATGGTCTTCGCTCCTCTT | Yes |
| Glyma19g41280.1 | GmNC2α7 | CACTCGCTGTGCCTGTTTTA | GAACTCATAGGGCAAATGCAA | No |
| Glyma05g07750.1 | GmNC2β1 | CACCTCCTCTTCCTTCCACA | AGCGTCTTCCTTTGACTTGC | Yes |
| Glyma06g23234.1 | GmNC2β2 | TGCAAGATTCTTTAAAAGGTGCA | GCCTCTGGCTCCTTGGATTG | Yes |
| Glyma17g13260.1 | GmNC2β3 | CAGTGTGGGTTGCCCTAATC | AGGAGGTGCAAGAGCGAATA | Yes |
| Glyma18g22896.1 | GmNC2β4 | TGCAAGATTCTTTAAAAGGTGCA | GCGTCTGGCTGCTTGGAAGT | Yes |
| Glyma11g37130.1 | GmDpb3-1 | GCTGACGAAGAAGAAAACGG | TTTCAGCCAGAAATTGGAGG | Yes |
| Glyma18g01040.1 | GmDpb3-2 | TCCGGAAACCAGAACTTGAC | CATCTACGGGTACTGGCGAT | Yes |
| Glyma02g35190.4 | GmNF-YA01 | CACTCTCTAGCAGACATGATAGGTA | ACATCGCAGCACCAAGCCCAC | Yes |
| Glyma02g47380.5 | GmNF-YA02 | TCACAACTGAAGTGAGATATCAA | AGCCCCCCAAAGAGAATGAA | Yes |
| Glyma03g36140.5 | GmNF-YA03 | AAAGAATCAAGGGTGGGCTT | CCAAGGATGGATTGCCTAGA | Yes |
| Glyma05g29970.1 | GmNF-YA04 | CTTTTACCTCTCTATATTCTGGCT | CCATTTACAGATGGGGGCTGC | Yes |
| Glyma07g04050.9 | GmNF-YA05 | GTGTGGAAGGTGTCATCGG | AATATTAGATTGTGGCACAAATG | Yes |
| Glyma08g13090.1 | GmNF-YA06 | GGCTTCTGTTCTTTCTACCCA | CAGTAAGGTCGTGAGCAGAG | Yes |
| Glyma08g45030.1 | GmNF-YA07 | ACCTGGCGGTGTTTATTCTG | TATTGGACAAAGACTGGCCC | Yes |
| Glyma09g02770.2 | GmNF-YA08 | GGATAGGTGGCAGCTACGACACAACA | ATTGAACTGAAGTGTTGCTGC | Yes |
| Glyma09g07960.4 | GmNF-YA09 | GGCGATCCAATTTTTGCTTA | GTATACCATGGTATTGTTTTGCA | Yes |
| Glyma10g10240.2 | GmNF-YA10 | CAACTACGTCGACCAATTCCCA | GGTAAACTGGGTTGTTGTATG | Yes |
| Glyma12g36540.6 | GmNF-YA11 | TCCACTTCAGACAAAGGCAAC | CCTATAGTGAAACTCTGCTCTG | Yes |
| Glyma13g16770.1 | GmNF-YA12 | TTATTTATGGACTCTGCGTGC | GCATGTCTCCTCTGATAAGCC | Yes |
| Glyma13g27230.4 | GmNF-YA13 | CAGACATTGGGCAAAATACTGG | CCAATATTGAAACTCTCCACTC | Yes |
| Glyma14g01360.1 | GmNF-YA14 | TTGGGGGATTTTGAGGATTAG | GGAGCCGCAGCTGTTTAAAGGT | Yes |
| Glyma15g03175.1 | GmNF-YA15 | CTCGAAGCGCTTAACAAACC | ATTGAGAAAGCGTCCACCAG | Yes |
| Glyma15g13660.2 | GmNF-YA16 | TTGCAGCAGCACTTCAATTC | CAAAGGGTGGTTTTCGGTTA | Yes |
| Glyma15g18970.1 | GmNF-YA17 | GGCGATCCAATTCTTGCTTA | CATGGTATTGTTTCGCGTTG | Yes |
| Glyma16g00711.1 | GmNF-YA18 | TAGTCTGTTTACAGGAAGTAATGT | AAGACAAGTCAGCTAGTGCTAA | Yes |
| Glyma17g05920.1 | GmNF-YA19 | ACGCATTGAACAGGGTTAGG | TTGAGGGATGATTTTCTGCC | Yes |
| Glyma18g07890.2 | GmNF-YA20 | CAAATGCATCCAATTCATCG | TTCCAGCTGTGGTGTCTGAG | Yes |
| Glyma19g38800.2 | GmNF-YA21 | GCTTTTGGATCACAATCCGT | CTTGCTCAGCTCCTCTTGCT | Yes |
| Glyma02g17310.1 | GmNF-YB01 | CATATAAATTAATTATGTGG | CTTGAGTATCTCAAATTCAGGA | Yes |
| Glyma02g46970.1 | GmNF-YB02 | CACTTATCCTGCCCCTGGTA | AACCAACCCAGTGATCTTGC | Yes |
| Glyma03g18670.2 | GmNF-YB03 | CGAAGGAGACAATCCAGGAG | GCGATAGCGGTGAAGGTAAA | Yes |
| Glyma03g22721.1 | GmNF-YB04 | GGAGTGCGTGTCAGAGTTCA | GGGTGTGAGAGTTTCTGGGA | Yes |
| Glyma03g33490.1 | GmNF-YB05 | AGAAGCGAAAAGCGAGTGAG | AGAAAGCAAGGTGGAGACGA | Yes |
| Glyma05g31681.1 | GmNF-YB06 | TCTCAAAGGAAGCCAAGGAA | TAGTGTTGTCATGGCCCAAA | Yes |
| Glyma05g32680.1 | GmNF-YB07 | AAAAAGTGATTCCCCCCAAC | TCTTTTCCCTTTGGCATTTG | Yes |
| Glyma07g29695.1 | GmNF-YB08 | GGATCGATGCCAAAGTGAGT | AGTGAGAGGCTCAACGTAGTCA | No |
| Glyma07g37840.2 | GmNF-YB09 | GTAACGAGCGAGGCGTCA | CTATTTCCTCTATCGACTTCTA | Yes |
| Glyma07g39820.1 | GmNF-YB10 | TCTCCGATGATGCAAAGGAG | GTCGAATCCAAGCTTACTCATT | Yes |
| Glyma08g00330.2 | GmNF-YB11 | TAACAATAATCATCAGAACCATAGC | CCTGTCACAAAACTTATGAACTCG | Yes |
| Glyma08g14931.1 | GmNF-YB12 | CAGCCAATGCAAAAATCTCA | TTTCCCTCTGGCACTTGTCT | Yes |
| Glyma08g44140.2 | GmNF-YB13 | TCCCACACAAAATTGCACGATTCTG | TGAATGAGAAGTTGAAAATTCGAGTC | Yes |
| Glyma09g01650.1 | GmNF-YB14 | CAGGTTCCTCCCAATAGCAA | TTCCTCTTCTCCTTCTGGCA | Yes |
| Glyma09g05150.2 | GmNF-YB15 | ACAACTTTAACATGTGCCAAGCA | TGCTATCTTCCATTGATTTTTTGAT | Yes |
| Glyma09g28671.1 | GmNF-YB16 | ACGAGTCCAGGCAATGGTAG | GAACAGTCTCTTTGGCGTCC | Yes |
| Glyma10g02480.1 | GmNF-YB17 | CATATAAATTAATTAGGGAA | CTTGAATATATGTCAGGAGATC | Yes |
| Glyma10g05606.1 | GmNF-YB18 | TTCATCAGCTTCGTCACCAG | CTGTAAGCGGCGAGGTAAAC | Yes |
| Glyma10g29440.2 | GmNF-YB19 | CTAATCAATCTGGATGAGTACGT | TGTCAGTCCCCACTCCCTC | Yes |
| Glyma10g33550.2 | GmNF-YB20 | TCATCCCTTCCCCTTTCTCT | CGCCACCACTCTCGTGAC | Yes |
| Glyma11g18190.1 | GmNF-YB21 | ACTCCGACAATGACTCCGAC | TACTCCTGCACCGTCTCCTT | Yes |
| Glyma11g29866.1 | GmNF-YB22 | CGAACACAGTCCTCACTCCA | CTGATGAACTCGAAGACGCA | No |
| Glyma13g10690.2 | GmNF-YB23 | TCCTTCCAACAGGAAAATCG | CGTTGATGGTCTTCCTCTTCTC | No |
| Glyma15g12570.1 | GmNF-YB24 | CGTGAGCAGGATCATGAAGA | GCACTTATCGGACGCTTCTC | Yes |
| Glyma15g16460.2 | GmNF-YB25 | CGACAACAACAACAATGGTGGC | CACACATTCCTGCATCGTCTCC | Yes |
| Glyma17g00950.1 | GmNF-YB26 | TCTCGGACGATGCAAAAGAA | GTCGAATCCAAGCTTGCTCATG | Yes |
| Glyma17g02810.1 | GmNF-YB27 | GTAACCAGCGAGGCGTCG | GTTATTTGCTCTATCTAGTTCTT | Yes |
| Glyma18g08620.1 | GmNF-YB28 | AGCCTCTCAAGGGCTACCTC | ATCATGATTCCACCAGGAGC | Yes |
| Glyma19g36220.2 | GmNF-YB29 | GTACCTCCCCATCGCTAACA | CTCGCTGGTGATGAAACTGA | Yes |
| Glyma20g00240.1 | GmNF-YB30 | CTTCTGCAATGGGGATCAAT | TCGTCCCTGTAAAACCCTTG | Yes |
| Glyma20g34050.2 | GmNF-YB31 | GTGAGATGTCGGATGCGCCAC | CGGCTGATGTTGGCAATCGGG | Yes |
| Glyma20g37870.2 | GmNF-YB32 | ACAAATCAATCTGGATGAAAGTATA | TGATACACTACTTAAAGAAGATAG | Yes |
| Glyma02g09867.1 | GmNF-YC01 | TCACTTTTCGTGCTTGGATG | GCCGAGGTTGTTTGGAATAA | Yes |
| Glyma03g39911.1 | GmNF-YC02 | GGAGCGCAGCGTCATCTTTTA | CCAGCAGGTTGACCGGTGG | Yes |
| Glyma04g37291.1 | GmNF-YC03 | GCAGGTGCAGGCGCGGC | ATCTTCTTGATGCGGGCCAAG | Yes |
| Glyma06g17780.1 | GmNF-YC04 | AAAATTGGGGGAATGGAGAC | CTGGAGAAGGTGCTGGAAAG | Yes |
| Glyma08g15700.1 | GmNF-YC05 | AGCATGGTCAACCAACACAA | GTCTTGGGGAGCATGTTGTT | Yes |
| Glyma08g17630.1 | GmNF-YC06 | CGTCCATTGCAAAGTCTTGC | CCTGCGGCAACTCCCATCA | Yes |
| Glyma10g29691.1 | GmNF-YC07 | AATGATATCGGCTGAGGCAC | TCAGTTCTTGTGATGGCTGC | Yes |
| Glyma12g34510.1 | GmNF-YC08 | ATCACTTTGGTTTCCAATTCTGAC | TAGTTATGTCACACACACACACAC | No |
| Glyma13g27770.2 | GmNF-YC09 | GGCTTCAGGTAGTGCTGAGG | CAAACCCTTGTTGCTCCATT | Yes |
| Glyma13g27780.2 | GmNF-YC10 | TTGGGCTAATGTTGAGGAGG | GGCCTACAAACCCTTGTTGA | Yes |
| Glyma13g27790.1 | GmNF-YC11 | AAGGTGAAGCAGCAGAAGGA | TTGGGAGGATTTGTAGGTGC | Yes |
| Glyma13g35980.1 | GmNF-YC12 | CCAAGGCTTGTGAGCTCTTC | CAACTGCATGGCTTTCAGAAC | Yes |
| Glyma15g41486.1 | GmNF-YC13 | TCCATCGCAAAGGAAGTCGC | CTGTGGCAACGCCCATCC | No |
| Glyma19g42460.1 | GmNF-YC14 | AAGCGCTGCATCATCCTTTG | TGAGCTCCAGCAGGTGTGG | Yes |
| Glyma20g37620.1 | GmNF-YC15 | GCAGCCATCACAAGAACTGA | TGTGCATGTTGAGGTGGAAT | Yes |
