# Supplementary material for: Genome-wide expression analysis of soybean NF-Y genes reveals potential function in development and drought response
Source: Mol Genet Genomics. 2014 Dec 27;290(3):1095–115. doi: 10.1007/s00438-014-0978-2 (PMC4435856; doi:10.1007/s00438-014-0978-2)
Supplement: Supplementary file 5 — Supplementary material 5 (DOCX 21 kb) [file 438_2014_978_MOESM5_ESM.docx]

Supplementary Table S3 Reference genes used to determine the best reference gene combination in the real time PCR analysis for drought response.

Genome-Wide Expression Analysis of Soybean NF-Y Genes Reveals Potential Function in Development and Drought Response

Truyen N Quach^1,2 §^, Hanh TM Nguyen^1,3 §^, Babu Valliyodan^1^, Trupti Joshi^4^, Dong Xu^4^, Henry T. Nguyen^1*^

^1^Division of Plant Sciences, National Center for Soybean Biotechnology, University of Missouri, Columbia, MO, USA

^2^Current address: Field Crop Research Institute, Vietnam Academy of Agricultural Sciences, Hanoi, Vietnam

^3^Current address: The Center for Plant Science Innovation, University of Nebraska, Lincoln, NE, USA

^4^Department of Computer Science, Christopher S. Bond Life Sciences Center, National Center for Soybean Biotechnology and Informatics Institute, University of Missouri, Columbia, MO, USA

^§^These authors contribute equally to the research

^*^Corresponding author:

Henry T. Nguyen

National Center for Soybean Biotechnology and Division of Plant Sciences, University of Missouri, Columbia, Missouri 65211, USA.

Tel: 573-882-5494

Fax: 573-882-1469

E-mail: [nguyenhenry@missouri.edu](mailto:nguyenhenry@missouri.edu)

| **Gene symbol** | **Function** | **NCBI Accession/**  **Plant GI** | **Forward primer sequence [5'-3']** | **Reverse primer sequence [5'-3']** | **Reference** |
| --- | --- | --- | --- | --- | --- |
| **ACT11** | Cytoskeletal structural protein | BW652479 | ATCTTGACTGAGCGTGGTTATTCC | GCTGGTCCTGGCTGTCTCC | ([Hu, Fan et al. 2009](#_ENREF_1)) |
| **TIP41** | TOR (Target of Rapamycin) signalling element | EV263725 | AGGATGAACTCGCTGATAATGG | CAGAAACGCAACAGAAGAAACC | ([Hu, Fan et al. 2009](#_ENREF_1)) |
| **TUB4** | Structural constituent of cytoskeleton | EV263740 | GGCGTCCACATTCATTGGA | CCGGTGTACCAATGCAAGAA | ([Hu, Fan et al. 2009](#_ENREF_1)) |
| **UKN1** | Unkown | BU578186 | TGGTGCTGCCGCTATTTACTG | GGTGGAAGGAACTGCTAACAATC | ([Hu, Fan et al. 2009](#_ENREF_1)) |
| **UKN2** | Unkown | BE330043 | GCCTCTGGATACCTGCTCAAG | ACCTCCTCCTCAAACTCCTCTG | ([Hu, Fan et al. 2009](#_ENREF_1)) |
| **CYP2** | cyclophilin | TC224926 | CGGGACCAGTGTGCTTCTTCA | CCCCTCCACTACAAAGGCTCG | ([Jian, Liu et al. 2008](#_ENREF_2)) |
| **IDE** | Insulin degrading enzyme | AW310136 | ATGAATGACGGTTCCCATGTA | GGCATTAAGGCAGCTCACTCT | ([Libault, Thibivilliers et al. 2008](#_ENREF_3)) |
| **SUBI-2** | Ubiquitin | D26092 | AGCTATTCGCAGTTCCCAAAT | CAGAGACGAACCTTGAGGAGA | ([Libault, Thibivilliers et al. 2008](#_ENREF_3)) |
| **CDPK** | CDPK-related protein kinase | AW396185 | TAAAGAGCACCATGCCTATCC | TGGTTATGTGAGCAGATGCAA | ([Libault, Thibivilliers et al. 2008](#_ENREF_3)) |

Hu, R., C. Fan, et al. (2009). "Evaluation of putative reference genes for gene expression normalization in soybean by quantitative real-time RT-PCR." BMC Mol Biol **10**: 93.

Jian, B., B. Liu, et al. (2008). "Validation of internal control for gene expression study in soybean by quantitative real-time PCR." BMC Mol Biol **9**: 59.

Libault, M., S. Thibivilliers, et al. (2008). "Identification of four soybean reference genes for gene expression normalization." The Plant Genome **1**(1): 44-54.
